# Effect of enteral arachidonic acid and docosahexaenoic acid supplementation on brain volumes at term in preterm infants: a secondary outcome analysis of a randomised controlled trial

**DOI:** 10.1136/archdischild-2024-328292

**Published:** 2026-01-19

**Authors:** William Hellström, Pia Lundgren, Anders K Nilsson, Staffan Nilsson, Anna-Lena Hård, Ulrika Sjöbom, Chatarina Löfqvist, Isabella M Björkman-Burtscher, Dirk Wackernagel, Ingrid Hansen-Pupp, Lois EH Smith, Boubou Hallberg, Karin Sävman, David Ley, Ann Hellström, Rolf A Heckemann

**Affiliations:** 1 Institute of Clinical Sciences, Department of Pediatrics, University of Gothenburg, Sahlgrenska Academy, Gothenburg, Sweden; 2 Region Västra Götaland, Dept of Pediatrics, The Queen Silvia Children′s Hospital, Sahlgrenska University Hospital, Gothenburg, Sweden; 3 Section for Ophthalmology, Institute of Neuroscience and Physiology, Sahlgrenska Academy at University of Gothenburg, Gothenburg, Sweden; 4 Region Västra Götaland, Dept of Pediatric Ophtalmology, The Queen Silvia Children′s Hospital, Sahlgrenska University Hospital, Gothenburg, Sweden; 5 Institute of Biomedicine, Sahlgrenska Academy, University of Gothenburg, Gothenburg, Sweden; 6 Institute of Health and Care Sciences, University of Gothenburg Sahlgrenska Academy, Gothenburg, Sweden; 7 Department of Radiology, Institute of Clinical Sciences, Sahlgrenska Academy, University of Gothenburg and Sahlgrenska University Hospital, Gothenburg, Sweden; 8 Department of Radiology, Section of Neuroradiology, Sahlgrenska University Hospital, Region Västra Götaland, Gothenburg, Sweden; 9 Department of Clinical Science, Intervention and Technology (CLINTEC), Karolinska Institutet, Stockholm, Sweden; 10 Division of Neonatology, Department of Pediatrics, University Medical Center of the Johannes Gutenberg-University Mainz, Mainz, Germany; 11 Department of Clinical Sciences, Lund, Pediatrics, Lund University, and Skåne University Hospital, Lund, Sweden; 12 The Department of Ophthalmology, Boston Children’s Hospital, Harvard Medical School, Boston, Massachusetts, USA; 13 Sahlgrenska Academy, University of Gothenburg, Gothenburg, Sweden; 14 Region Västra Götaland, Dept of Neonatology, The Queen Silvia Children′s Hospital, Sahlgrenska University Hospital, Gothenburg, Sweden; 15 Department of Medical Radiation Sciences, Clinical Sciences, Sahlgrenska Academy, University of Gothenburg, Gothenburg, Sweden

**Keywords:** Neonatology, Magnetic Resonance Imaging, Infant Development, Therapeutics

## Abstract

**Objective:**

Investigate whether enteral supplementation with arachidonic acid (AA) and docosahexaenoic acid (DHA), from birth to term-equivalent age (TEA), promotes brain maturation as a prespecified secondary outcome of a multicentre randomised controlled trial.

**Participants:**

206 infants born at 22–28 weeks gestational age (GA) were randomised into intervention or control groups from three university hospitals in Sweden.

**Intervention:**

The intervention group received an oil with AA (100 mg/kg/d) and DHA (50 mg/kg/d) starting at birth until 40 weeks postmenstrual age (PMA) in addition to standard nutrition. Standard-of-care infants received standard nutrition according to national guidelines.

**Main outcome and measures:**

MRI volumetrics were defined *a priori* as a secondary outcome of the trial and included total brain, white and cortical grey matter, central structures and cerebellum. Univariable and multivariable linear regression models were used for comparisons.

**Results:**

MRI data in 117 infants had sufficient quality for inclusion (n=58 intervention). Birth weight, GA at birth, sex distribution, and PMA at MRI were similar in the groups. Infants receiving intervention had significantly larger white-matter volume at TEA, as compared with standard of care, in models adjusted for GA at birth, sex, study centre and PMA at MRI (β=6.8 cm^3^, 95% CI 0.7 to 12.9, p=0.028). The contribution of the intervention to white-matter volume corresponded to 10 days of prolonged gestation.

**Conclusion and relevance:**

Our findings in this hypothesis-generating study suggest that AA+DHA promotes white matter growth, which may protect the developing brain in this vulnerable population.

**Trial registration number:**

NCT03201588.

**WHAT IS ALREADY KNOWN ON THIS TOPIC**

- Extremely preterm infants (born <28 weeks of gestation) are particularly susceptible to altered brain maturation and impaired neurodevelopment.
- Omega-3 and omega-6 long-chain polyunsaturated fatty acids such as arachidonic acid (AA) and docosahexaenoic acid (DHA) have critical roles in brain development and function.
- Neither lipid solutions for parenteral use, nor human breast milk contain sufficient amounts of AA:DHA to meet the accretion rate after extremely preterm birth.

**WHAT THIS STUDY ADDS**

- Data are sparse on the role of enteral supplementation of AA:DHA (100 mg/kg/d:50 mg/kg/d) on brain volumes at term-equivalent age in extremely preterm infants
- Extremely preterm infants who received supplementation with AA and DHA had significantly larger white matter volumes at term-equivalent age compared with standard-of-care infants.

**HOW THIS MIGHT AFFECT RESEARCH, PRACTICE OR POLICY**

- Our findings suggest that supplementation with AA+DHA in extremely preterm infants promotes brain growth and may thus be a strategy to promote brain maturation and neurodevelopment.

## Introduction

 Infants born extremely preterm with gestational age (GA) <28 weeks are particularly susceptible to brain injuries and altered brain maturation following birth, resulting in impaired neurodevelopment.^1–4^ The aetiology is only partly accounted for by macroscopic brain injuries such as intraventricular haemorrhage (IVH). Instead, the dominant underlying pathology is likely preterm-related microstructural alterations of the immature brain, with glia involvement accompanied by impaired axonal development, leading to altered myelination and brain growth patterns.^5 6^ Infants born extremely preterm generally have smaller grey- and white-matter volumes at term, with white-matter losses noted later in life.^4 7^ Deficits in white-matter volumes and signs of previous macroscopic injuries at term-equivalent age (TEA) have been found to be associated with unfavourable neurodevelopmental outcomes.^8–10^

The impact of nutrition on the development of the human brain, particularly in vulnerable populations such as preterm infants, has been the subject of extensive research and clinical interest. Among the various dietary components, omega-3 and omega-6 long-chain polyunsaturated fatty acids (LC-PUFAs) have attracted much attention due to their critical roles in brain growth and function.^11 12^

During gestation, the placenta selectively transfers the omega-6 LC-PUFAs arachidonic acid (AA, 20:4 n-6), adrenic acid (22:4 n-6) and other omega-6 allies and, to a lesser degree, the omega-3 fatty acid docosahexaenoic acid (DHA, 22:6 n-3) to the fetus.^11^ AA is involved in a multitude of physiological processes such as vascularisation, growth, immune regulation and reproduction.^11^ AA is the main omega-6 LC-PUFA in the brain.^13^ DHA is the only omega-3 LC-PUFA present in significant amounts in the brain, especially abundant in neurons and retinal rod outer segments in particular.^13 14^ DHA is crucial for neurodevelopment, synaptic plasticity and neuroinflammation regulation, and higher serum levels of DHA during early postnatal development were positively associated with several regional brain volumes at TEA in extremely preterm infants.^12^

The estimated fetal accretion during the third trimester of AA and DHA is 212 mg/kg/d and 43 mg/kg/d, respectively.^15^ Neither lipid solutions for parenteral use nor human breast milk contain sufficient amounts of AA and DHA to meet the needs after extremely preterm birth.

It has been hypothesised that supplementation with LC-PUFAs to preterm infants might promote brain growth and function. In randomised trials, infants born very preterm receiving formula milk with an AA:DHA ratio 2:1 had superior results at neurodevelopmental tests at 24 months of age as compared with infants receiving AA:DHA ratio 1:1,^16^ and in a randomised trial exploring the role of high DHA supplementation, Bayley mental developmental index (MDI) scores did not differ between groups; however, girls fed with high DHA had a higher MDI score as compared with standard nutrition.^17^ In summary, its effect on neurodevelopmental outcomes has yielded inconsistent results partly due to methodologic differences between studies.^18^

The present study was a prespecified secondary outcome of the Mega Donna Mega (MDM) trial evaluating the effect of enteral supplementation with AA (100 mg/kg/d) and DHA (50 mg/kg/d) from birth to TEA on retinopathy of prematurity (ROP) and other morbidities in infants <28 weeks GA. The supplementation was associated with a 50% reduction in severe ROP,^19^ and low visual acuity was less common at 2.5 years of age in children who received neonatal supplementation in the sensitivity analysis.^20^ In a smaller cohort of infants (<29 weeks GA), the same supplementation as in the present study was associated with improved white matter maturation, as assessed by diffusion tensor imaging.^21^

In this study, we compared brain volumes at TEA in infants with and without AA-DHA supplementation in the MDM trial.

## Methods

### Study design

This study reports on a secondary outcome of the randomised, open-label clinical trial MDM including infants recruited between December 2016 and August 2019 at three level III neonatal intensive care units in Sweden: The Queen Silvia Children’s Hospital, Gothenburg, Karolinska University Hospital, Stockholm, and Skåne University Hospital, Lund. MRIs were acquired between May 2017 and December 2019.

The primary outcome of the trial was the frequency of severe ROP, reported previously.^19^ Brain development/morphology determined by MRI at TEA was a predefined secondary outcome.

Eligible for inclusion were infants born between 22 and 28 weeks GA without detectable clinical gross malformations, syndromes, therapies or diseases that, according to the investigators’ opinion, made them unsuitable for participation in the trial (online supplement).

Infants in the intervention arm received a daily dose of a triglyceride oil (Formulaid®, DSM Nutritional Products Ltd, Switzerland) providing AA (100 mg/kg) and DHA (50 mg/kg). The intervention was started within 3 days after birth and lasted until TEA. The dose (0.1–1.0 mL) was adjusted weekly according to body weight (online supplement). Infants in the standard-of-care arm received standard nutrition (no placebo was administered). Further details of the intervention and regarding inclusion and safety data have been published.^19 22^ Additional information on nutritional strategies, randomisation, inclusion criteria is available in the online supplement. The study was approved by the Regional Ethical Review Board, University of Gothenburg (Dnr. 303–11, T570-15).

### MRI acquisition and volumetric segmentation

MRI acquisition was performed at TEA using 3.0-T systems: a GE DISCOVERY MR750w (GE Medical Systems, Waukesha, WI) at Queen Silvia Children’s Hospital (Gothenburg), a Siemens Prisma 3T system (Siemens Medical Systems, Malvern, PA, USA) at Skåne University Hospital (Lund) and a GE DISCOVERY MR750w at Karolinska University Hospital (Stockholm). The scanning protocols followed local clinical routines. Scanning protocols are presented in Supplement. For inclusion in data analysis, MRI had to be performed no later than at postmenstrual age (PMA) 46+6 weeks.

Brain volumetry was enabled by automatic anatomical segmentation using the DrawEM module of the Medical Image Registration Toolkit.^23^ DrawEM implements an atlas-based approach; the atlas database used consisted of 20 neonatal T2-weighted head images with corresponding anatomical label sets prepared by human experts.^24 25^

DrawEM accepts 3D T2 image volumes as input. Image volumes of the required format, termed ‘image stack’, were created from each acquisition (2D and direct 3D). Additional information on the segmentation process is available in the Supplement. Merged volumes of brain regions (total brain (ie, total intracranial volume without cerebrospinal fluid and ventricles), white matter, cortical grey matter, central structures and cerebellum) were generated by summation of selected individual regions as previously reported.^26^

### Infant characteristics

Birth characteristics and neonatal morbidities included the presence of ROP, necrotising enterocolitis (NEC, Bell’s stage 2–3), and bronchopulmonary dysplasia (BPD, oxygen need at 36 weeks). Severe IVH was classified according to modified Papile criteria (online supplement^27^). The Fenton growth chart was used to determine weight z-scores.^28^

### Statistics

Statistical analyses were performed in IBM SPSS Statistics (V.29.0.0.0, IBM, Armonk, NY) and R (version 4.3.1, R Foundation for Statistical Computing, Vienna, Austria). Data visualisation was done with RStudio^29^ and the ggplot2 package.^30^

Independent variables in the initial univariable analysis were GA at birth, birth weight SD score (BWSDS), sex (female reference), severe IVH, PMA at the time of MRI and centre (centre three reference). In the final multivariable analysis, GA at birth, sex, PMA at the time of MRI and centre were included. The primary statistical outcomes of the study are listed in the Supplement.

The model was run on total brain volume, following model validation in subregions for eligibility. Included variables in the regression models were checked for multicollinearity accompanied by visual inspection. For group comparisons of non-normally distributed variables and categorical variables, the Mann-Whitney *U* test, *χ*^2^ test of independence or Fisher’s exact test were used. In all analyses, the significance level was 0.05.

## Results

### Cohort description

**Figure 1:**
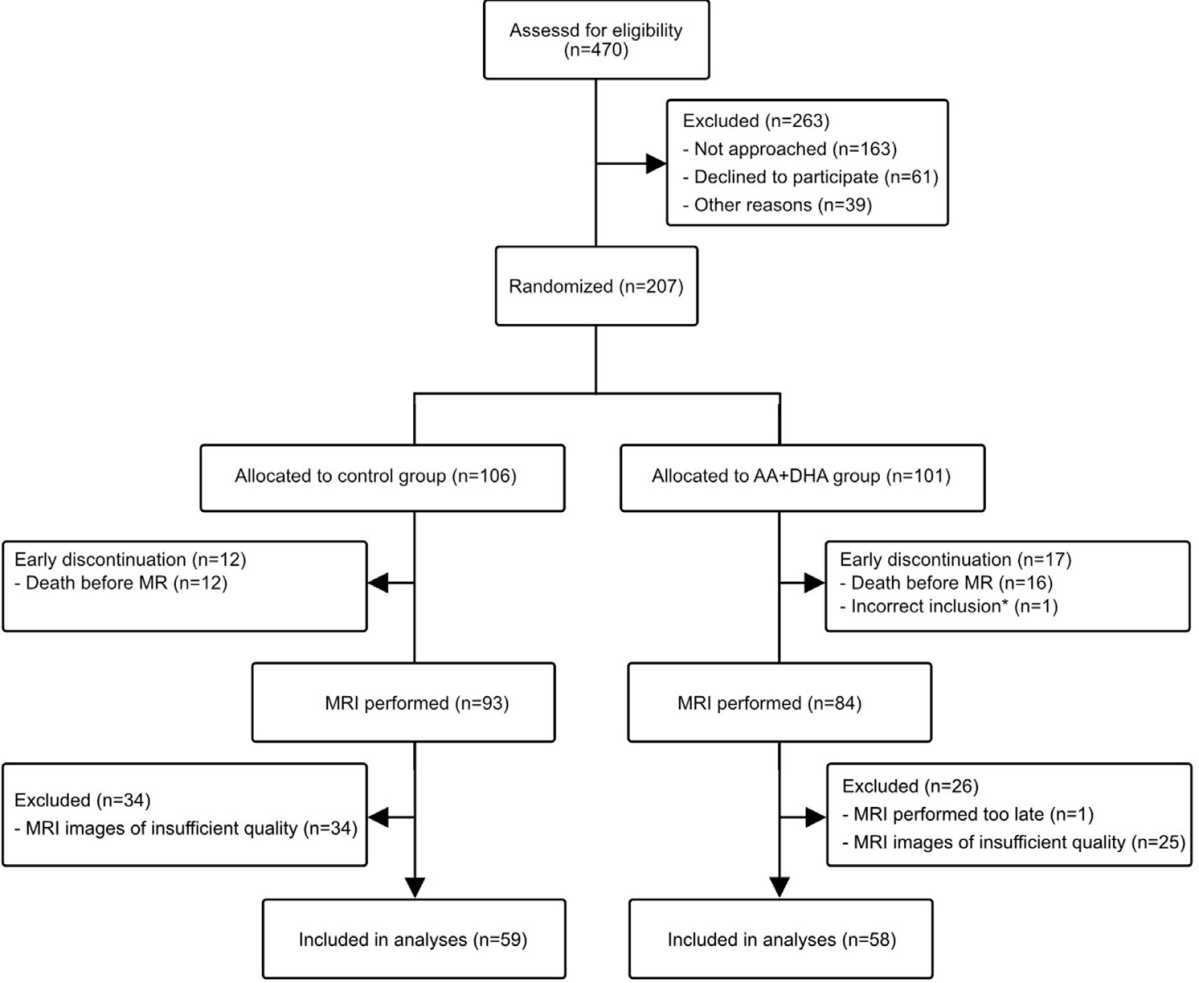
Consort flow diagram. AA, arachidonic acid; DHA, docosahexaenoic acid.

Of 206 infants randomised in the original trial, 177 underwent MRI at TEA (n=93 standard of care and n=84 intervention). One infant was excluded as the MRI was performed outside the acceptable time frame, and data from 59 infants were of insufficient quality for volumetric determination, leaving 117 infants in the final analyses (n=59 standard of care and n=58 intervention). The majority (63%) of the excluded infants were from centre 2 (table 1). A flow chart of the cohort is shown in figure 1.

**Table 1:** Birth characteristics, neonatal morbidities and nutritional support in included infants by intervention group and excluded infants

	All included infants (n=117*)*	Standard of care (n=59)	AA+DHA (n=58)	P value (standard of care vs AA+DHA)	Excluded infants (n=60)
Birth characteristics					
GA, weeks, mean (SD)	*25.6* (*1.5*)	25.5 (1.4)	25.8 (1.5)	0.27	*25.5* (*1.4*)
Male sex % (n)	*56% (65*)	56% (33)	55% (32)	0.93	*58% (35*)
BW, grams, mean (SD)	*808* (*199*)	787 (202)	830 (196)	0.16	*802* (*203*)
BWSDS (SD)	*0.10* (*0.80*)	0.05 (0.85)	0.16 (0.74)	0.51	*0.14* (*0.86*)
Twins, % (n)	*20% (23*)	22% (13)	17% (10)	0.64	*12% (7*)
Neonatal morbidities					
No IVH (%, no)	*60% (70*)	59% (35)	60% (35)	0.91	62% (37)
Severe IVH, % (n)	*11% (13*)	15% (9)	7% (4)	0.24	*13% (8*)
Severe ROP, > stage 3, % (n)	*24% (28*)	36% (21)	12% (7)	0.003	38% (23)*
NEC, % (n)	*4% (5*)	8% (5)	0	0.057	17% (10)**
BPD, % (n)	*26% (30*)	25% (15)	26% (15)	0.91	*37% (22*)
Nutritional support					
Energy intake (kcal/kg/day) 2–28 days, mean (SD)	*126.0* (*13.3*)	123.2 (12.8)	128.8 (13.4)	0.023	120.7 (15.2)*
Lipids intake (g/kg/day) 2–28, mean (SD)	*6.1* (*1.2*)	5.9 (1.2)	6.2 (1.2)	0.12	*5.8* (*1.4*)
Protein intake (g/kg/day) 2–28, mean (SD)	*3.8* (*0.4*)	3.8 (0.3)	3.9 (0.4)	0.75	3.6 (0.4)***
Carbohydrates intake (g/kg/day) 2–28, mean (SD)	*13.2* (*1.3*)	13.0 (1.0)	13.4 (1.5)	0.42	*13.0* (*1.2*)
Centre					
Centre 1	*43% (50*)	41% (24)	45% (26)	0.65	25% (15)***
Centre 2	*13% (15*)	14% (8)	12% (7)	0.81	63% (38)***
Centre 3	*44% (52*)	46% (27)	43% (25)	0.77	12% (7)***

For tests between two groups, Fisher’s exact test and Pearson’s *χ*2 test were used for dichotomous variables and the Mann-Whitney U-test for continuous variables.

In infants with MRI performed (n=177), significant differences between included and excluded infants (*variables in italics*) are marked with *p<0.05, **p<0.01 and ***<0.001.

AA, arachidonic acid; BPD, bronchopulmonary dysplasia; BW, birth weight; BWSDS, birth weight standard deviation score; DHA, docosahexaenoic acid; GA, gestational age; IVH, intraventricular haemorrhage; NEC, necrotising enterocolitis; ROP, retinopathy of prematurity.

**Figure 2:**
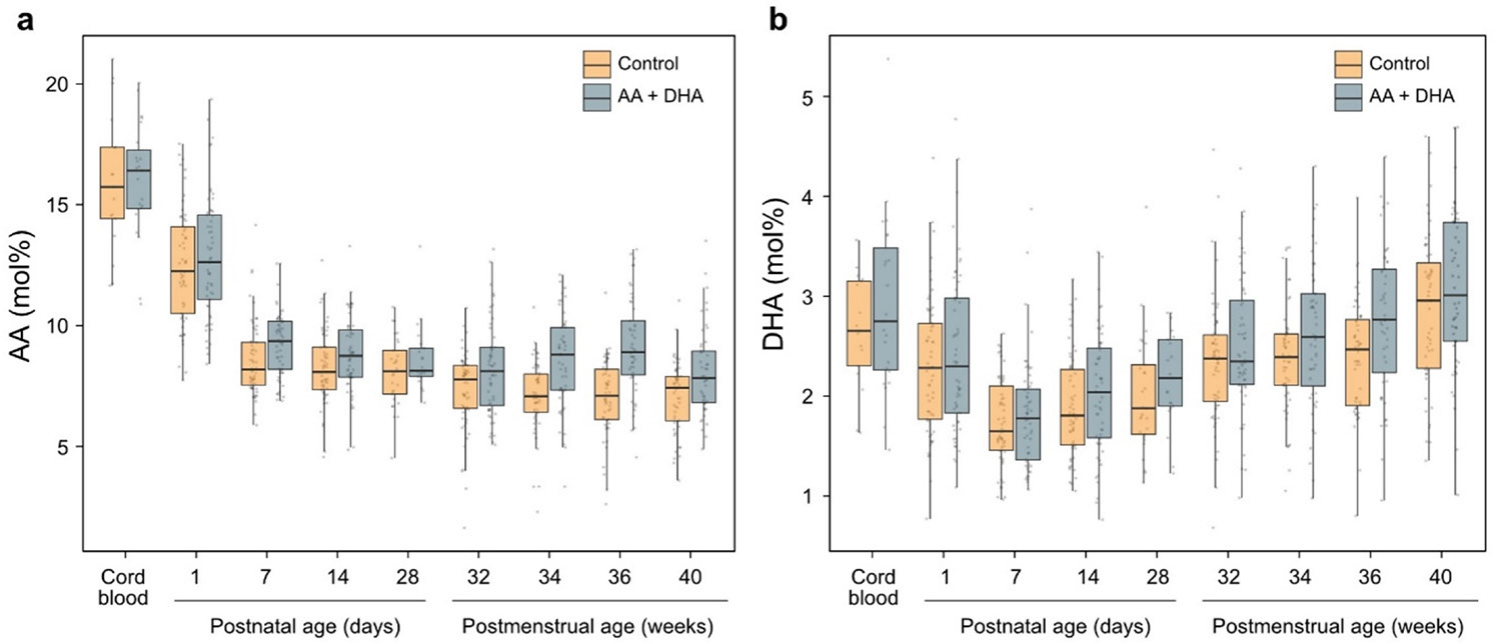
Postnatal serum levels of AA and DHA by postnatal age according to randomisation group. (a) Molar percentage (mol%) of AA and (b) molar percentage (mol%) DHA. Boxplots representing the IQR and whiskers Q1/Q3 -/+ 1.5 * IQR total range. Individual values are plotted centralised in boxplots in grey. Orange boxes represent the control group and grey the intervention group (AA+DHA). AA, arachidonic acid; DHA, docosahexaenoic acid.

Birth weight, GA at birth and sex distribution were similar between the standard of care and the intervention group (787 g vs 830 g, 25.5 weeks vs 25.8 weeks, males 56% vs 55%). There were no significant differences between the groups regarding severe IVH, NEC or BPD. However, infants in the intervention group were less affected by severe ROP, and in this follow-up cohort, 38% of the controls and 13% of the intervention infants developed severe ROP (p=0.003); these rates are similar to those reported for the complete cohort.^19^ Excluded infants (no T2 weighted MRI performed or poor MRI quality) had significantly more severe ROP and NEC, but similar rates of severe IVH and BPD as infants included in MRI analyses. Detailed infant characteristics by group are presented in table 1. The serum levels of AA and DHA (molar percentage) were elevated in infants receiving intervention as compared with standard of care group throughout the postnatal period until TEA, figure 2.

### Effect of the intervention on brain volumes at TEA

**Figure 3:**
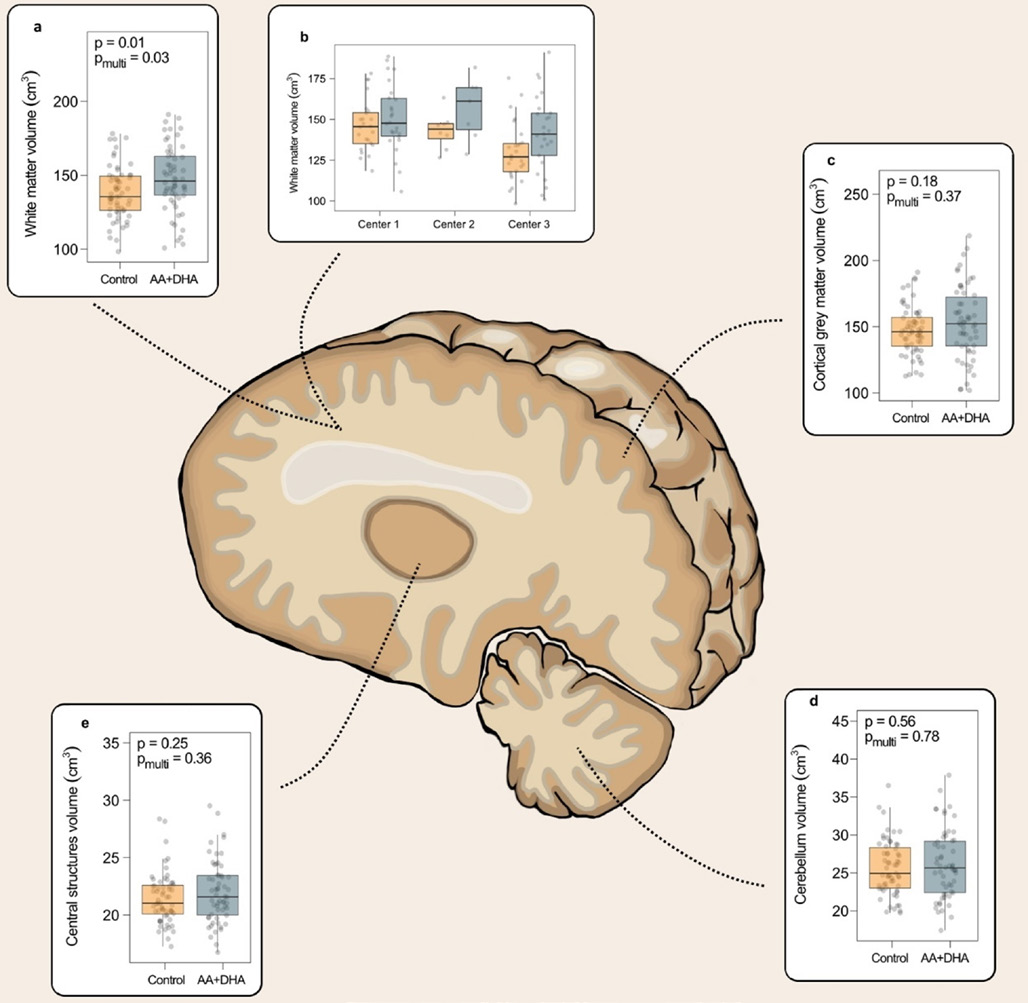
Total and regional brain volumes at term-equivalent MRI according to AA+DHA intervention group and standard-of-care. (a) White matter; (b) White matter. By centre, mean (SD) white-matter volume in cm^3^ in control group versus intervention group was 146.3 (16.0) versus 150.8 (20.8), 143.1 (11.2) versus 156.9 (18.8) and 129.7 (18.0) versus 141.8 (23.3) in centre 1, centre 2 and centre 3, respectively. (c) Cortical grey matter; (d) Cerebellum; and (e) Central structures. Dots represent individual values where n=59 for controls and n=58 for the intervention group (AA+DHA). Multivariable regression models were adjusted for gestational age at birth, sex, study centre and PMA at MRI. AA, arachidonic acid; DHA, docosahexaenoic acid; Pmulti, P value rendered by multivariable regression model.

Included MRI scans were acquired between PMA 37.6 and 46.4 weeks. There was no significant difference in PMA at MRI between the standard of care and intervention group, mean (SD) 41.4 (1.6) weeks versus 41.3 (1.3) weeks (p=0.38). Total and subregion brain volumes in standard-of-care and intervention infants are presented in figure 3. Brain volumes by randomisation group and centre are shown in online supplemental figure 1. Overall, similar trends between groups in brain volumetrics were seen at all three centres.

In univariable linear regression analysis, total brain volume was significantly higher in infants receiving the intervention (β=16.4, 95% CI 0.8 to 32.1 cm^3^, p=0.04); however, the significance did not persist in the multivariable analysis including PMA at time of MRI (weeks), GA at birth (weeks), sex and study centre (table 2).

**Table 2:** Brain volumes and univariable and multivariable linear regression for the association between MRI volumetrics (cm^3^) and variables

	Control	AA+DHA intervention (n=58)
	(n=59)	
Total brain volume (cm^3^), mean (SD)	341.6 (34.3)	358.1 (49.9)
White matter volume (cm^3^), mean (SD)	138.3 (18.0)	147.6 (22.1)
Cortical grey matter volume (cm^3^), mean (SD)	147.1 (18.4)	153.0 (27.6)
Central structures volume (cm^3^), mean (SD)	21.5 (2.2)	22.0 (2.7)
Cerebellum volumes (cm^3^), mean (SD)	25.7 (3.7)	26.1 (4.5)

Variable	Univariable linear regression			Multivariable linear regression		
	β	(95% CI)	P value	β	(95% CI)	P value
Total brain volume (cm^3^)						
AA+DHA intervention	16.4	0.8 – to 32.1	0.040	10.0	−1.7 to 21.8	0.092
GA at birth (weeks)	8.4	3.1 to 13.7	0.002	10.5	6.4 to 14.7	<0.001
Sex (males)	24.7	9.3 – to 40.1	0.002	25.3	13.5 to 37.1	<0.001
PMA at time of MRI (weeks)	15.2	10.4 to 19.9	<0.001	16.6	12.6 to 20.7	<0.001
Centre 1 (centre three reference)	6.0	−11.1 to 23.1	0.49	7.6	−5.2 to 20.3	0.24
Centre 2 (centre three reference)	10.6	−14.7 to 36.0	0.41	5.1	−13.4 to 23.6	0.58
Severe IVH	7.1	−18.2 to 32.5	0.58			
BWSDS	16.1	6.6 to 25.7	0.001			
White matter volume (cm^3^)						
AA+DHA intervention	9.4	2.0 to 16.7	0.013	6.8	0.7 to 12.9	0.028
GA at birth	4.0	1.5 to 6.5	0.002	4.9	2.8 to 7.1	<0.001
Sex (males)	11.8	4.5 to 19.1	0.002	10.6	4.5 to 16.8	<0.001
PMA at time of MRI	3.7	1.1 to 6.2	0.005	4.2	2.1 to 6.3	<0.001
Centre 1 (centre three reference)	13.1	5.4 to 20.8	0.001	14.1	7.4 to 20.7	<0.001
Centre 2 (centre three reference)	14.0	2.6 to 25.4	0.016	11.8	2.2 to 21.4	0.017
Severe IVH	2.7	−9.3 to 14.8	0.65			
BWSDS	6.8	2.2 to 11.4	0.004			
Cortical grey matter volumes (cm^3^)						
AA+DHA intervention	5.8	−2.8 to 14.4	0.18	3.1	−3.4 to 9.6	0.35
GA at birth	3.4	0.4 to 6.3	0.024	4.0	1.7 to 6.3	<0.001
Sex (males)	9.0	0.5 to 17.6	0.039	11.0	4.4 to 17.5	0.001
PMA at time of MRI	8.8	6.3 to 11.3	<0.001	9.5	7.2 to 11.7	<0.001
Centre 1 (Centre three reference)	−10.6	−19.7 to −1.5	0.022	−10.3	−17.4 to −3.2	0.005
Centre 2 (Centre three reference)	−6.6	−20.1 to 6.8	0.33	−9.0	−19.3 to 1.2	0.084
Severe IVH	2.0	−11.8 to 15.8	0.78			
BWSDS	7.6	2.3 to 12.8	0.005			
Central structures volume (cm^3^)						
AA+DHA intervention	0.5	−0.4 to 1.4	0.25	0.2	−0.6 to 1.0	0.60
GA at birth	0.4	0.06 to 0.7	0.018	0.5	0.2 to 0.8	<0.001
Sex (males)	1.2	0.3 to 2.1	0.007	1.1	0.4 to 1.9	0.008
PMA at time of MRI	0.6	0.3 to 0.9	<0.001	0.7	0.4 to 0.9	<0.001
Centre 1 (Centre three reference)	1.5	0.6 to 2.4	0.002	1.6	0.8 to 2.4	<0.001
Centre 2 (Centre three reference)	1.4	0.009 to 2.8	0.049	1.1	−0.06 to 2.3	0.063
Severe IVH	1.6	0.2 to 3.0	0.031			
BWSDS	0.6	0.003 to 1.1	0.049			
Cerebellum (cm^3^)						
AA+DHA intervention	0.4	−1.0 to 2.0	0.56	−0.2	−1.3 to 0.9	0.73
GA at birth	0.5	−0.02 to 1.0	0.059	0.8	0.4 to 1.2	<0.001
Sex (males)	1.9	0.4 to 3.4	0.012	1.9	0.9 to 3.0	<0.001
PMA at time of MRI	1.7	1.3 to 2.1	<0.001	1.9	1.5 to 2.2	<0.001
Centre 1 (Centre three reference)	1.1	−0.5 to 2.7	0.17	1.3	0.1 to 2.4	0.034
Centre 2 (Centre three reference)	1.3	−1.1 to 3.7	0.28	0.8	−0.9 to 2.5	0.34
Severe IVH	0.4	−2.0 to 2.8	0.74			
BWSDS	1.0	0.03 to 1.9	0.044			

The multivariable linear regression model included GA at birth, sex, PMA at MRI, and study centre.

AA, arachidonic acid; BW, birth weight; BWSDS, birth weight standard deviation score; DHA, docosahexaenoic acid; GA, gestational age; IVH, intraventricular haemorrhage; PMA, postmenstrual age.

Infants in the intervention group had significantly larger white matter volumes at TEA, both in univariable analysis (β=9.4, 95% CI 2.0 to 16.7 cm^3^, p=0.013), and multivariable analysis (β=6.8, 95% CI 0.7 to 12.9 cm^3^, p=0.028). The contribution of the intervention on white matter volume in the multivariable model corresponded to approximately 10 days of longer gestation in the present cohort. The positive effect of the intervention on white matter volume persisted when the presence of severe IVH was included in the multivariable model (β=7.2, 95% CI 1.0 to 13.3 cm^3^, p=0.022). The mean white-matter volumes in cm^3^ (SD) in the standard of care group versus the intervention group in centre 1, centre 2 and centre 3 were 146.3 (16.0) versus 150.8 (20.8), 143.1 (11.2) versus 156.9 (18.8) and 129.7 (18.0) versus 141.8 (23.3), respectively, whereas volumes of cortical grey matter, central structures and cerebellum volumes did not differ significantly (table 2). Additional data on regional brain volumes by study centre are shown in online supplemental table 1 and online supplemental figure 1.

### Nutritional intake and growth

There were no differences between standard-of-care and intervention infants regarding the intake of lipids, protein or carbohydrates during postnatal days 2–28. The mean energy intake during this period was lower among standard-of-care infants compared with intervention infants (123.2 kcal/kg/day vs 128.8 kcal/kg/day, p=0.023) (table 1). In multivariable analysis, no effect of mean energy intake on the association between intervention and white-matter volume could be shown (β=6,4, 95% CI 0.2 to 12.6 cm^3^, p=0.044). Following adjustment for BWSDS in the multivariable model for white-matter volume, results were unchanged (β=6.4, 95% CI 0.4 to 12.4 cm^3^, p=0.038). Furthermore, there were no differences between study groups in body weight at 36 or 40 weeks PMA or in change in weight z-score between birth and 36 or 40 weeks PMA (online supplemental table 2).

## Discussion

In this hypothesis-generating study, we report larger white-matter volume in extremely preterm infants after postnatal enteral supplementation with AA and DHA. Our results indicate enhanced white matter brain growth in supplemented infants corresponding to ~10 days longer gestation.

White matter disturbances following extremely preterm birth are the main underlying pathology of adverse neurodevelopment. Alterations in brain development trajectories in extremely preterm infants are partly suggested to be the result of a primary brain injury, followed by a secondary disturbance in programmed brain development.^31^ Myelination is initiated in the second half of gestation but mainly takes place after axonal overproduction and pruning after full-term birth.^32^ The development of the human brain is a complex, strictly hierarchical process of sequences including both structural and functional changes. Exposure to perinatal insults such as hypoxia/hyperoxia, insufficient nutrition and inflammation at a crucial developmental stage of pre-oligodendrocyte maturation into oligodendrocytes producing myelin results in preterm-related white-matter injuries, including signal abnormalities, volume deficit, thinning of the corpus callosum and delayed myelination.^31 33^

In general, total brain volume, as well as grey- and white-matter volume, is reduced at TEA in extremely preterm infants as compared with term infants and tightly related to immaturity.^7 34^ Reduction in white-matter volume in extremely preterm infants is reported to persist later in life.^4 35^ Further, white-matter volume at TEA is a major determinant of IQ in adolescents born preterm,^36^ and white-matter abnormalities, including loss of white-matter volume, are strongly associated with impaired cognitive development.^36^ Microstructural white-matter abnormalities are associated with motor and cognitive dysfunction later in life as well as autism spectrum disorder and attention deficit hyperactivity disorder, which are particularly common in children born extremely preterm.^33 37 38^

In both humans and animals, 50% of the adult amounts of AA and DHA accumulate in the brain during the period before myelination or when myelination has just started.^39^ In vitro, DHA and fish oil high in omega-3 LCPUFAs contribute to the proliferation, differentiation and maturation of oligodendrocyte precursor cells as well as to myelination.^40 41^ DHA counteracts the inhibition of oligodendrocyte precursor cell maturation induced by TNF-α, presumably through its PPAR-γ agonistic activity.^42^ In a mouse model of ROP, omega-3 PUFAs administered in the proliferative phase of the disease resulted in a PPAR-γ mediated direct anti-angiogenic effect.^43^ AA is the most abundant omega-6 fatty acid in the brain, particularly prevalent in endothelial cells and astrocytes.^11 13^ In addition, AA is the immediate precursor of adrenic acid which is an abundant PUFA in the brain and especially enriched in myelin. It is therefore likely that supplementation with AA and DHA after preterm birth, when the maternal supply is lost, may improve outcome.

A few studies have investigated the relationships between serum omega-3 and omega-6 fatty acids and brain volumes. Serum DHA levels have been positively associated with brain volumes at TEA in extremely preterm infants. AA levels, on the other hand, exhibited no such association.^12^ Supplementation of human milk with equal amounts of AA and DHA for approximately 9 weeks compared with placebo in infants with BW <1500 g resulted in improved cognition at 6 months^44^ and improved attention at 20 months.^45^ At 8 years of age, no significant effects on cognition, brain volumes, white matter microstructure or behaviour were found.^46 47^

In a recent trial, infants with GA <29 weeks received 100 mg/kg/day of AA and 50 mg/kg/day of DHA or medium chain triglycerides (standard of care) from the second day of life until 36 weeks’ PMA (n=60 per group).^21^ At TEA, diffusion tensor brain imaging indicated improved oligodendrocyte lineage maturation and myelination in the intervention group,^21^ but no volumetric results were reported. Similar to our study, the incidence of severe ROP was approximately 50% lower in the AA+DHA group, but the difference was not statistically significant, and the population was slightly more mature.^21^ Regarding ROP, we found that infants with GA <25 weeks benefitted more from the supplementation than more mature infants. This result again suggests that this subgroup of infants in particular benefits from supplementation in terms of brain growth and maturation.^19^

Aligning with our data indicating that AA+DHA supplementation concomitantly reduced severe ROP and increased white matter volumes, studies suggest a strong association between severe ROP and white matter volume, maturation and poorer neurodevelopment.^48 49^ In addition, substantially lower volumes of unmyelinated white matter at TEA and lower scores at Bayley Scales of Infant Development at 2 years were found in infants with any stage of ROP compared with no ROP.^50^

The association of ROP and whitematter abnormalities and the beneficial effect of AA+DHA supplementation on both outcomes indicates pathways in brain and retinal development amenable to common preventive strategies. Providing sufficient amounts and proper proportions of AA and DHA may prevent or mitigate central nervous system abnormalities after extremely preterm birth. Optimal dosage in relation to GA at birth, PMA and sex needs further study.

### Limitations and strengths

This study has limitations. The power calculations were based on severe ROP and not designed to demonstrate differences in pre-specified secondary outcomes. A proportion of infants did not have MRI scans of sufficient quality for volume segmentation, mainly due to incomplete follow-up and insufficient image quality. The baseline data of infants with available volumetric data did not, however, differ significantly from those without. Additional potential limitations include centre variability and a lower incidence of severe IVH in the treatment group. In this hypothesis-generating study, regression models were not adjusted for multiple testing. Although the MRI volumetric segmentation tool DrawEM was primarily validated in preterm infants and term-born neonates at TEA, we applied it in preterm infants scanned up to 6.4 weeks after TEA. Thorough visual inspection did not reveal increased segmentation failures; still, subtle effects on segmentation accuracy cannot be entirely ruled out. A strength of this study is the reported association between the intervention and circulating fatty acids and the reduction of ROP. Another strength is the robust association between intervention and white matter volume, regardless of study centre, despite potential differences in methodological and nutritional site strategies.

## Conclusion

In conclusion, this hypothesis-generating study shows that enteral supplementation with AA+DHA during the first months of life is associated with increased white-matter volume at TEA in extremely preterm infants. Our findings suggest that AA+DHA might promote white-matter development, which may protect the developing brain.

## Supplementary material

10.1136/archdischild-2024-328292 online supplemental file 1

10.1136/archdischild-2024-328292 online supplemental file 2

## Data Availability

No data are available.
