# Supplementary material for: Effect of enteral arachidonic acid and docosahexaenoic acid supplementation on brain volumes at term in preterm infants: a secondary outcome analysis of a randomised controlled trial
Source: Arch Dis Child Fetal Neonatal Ed. 2026 Jan 19;111(4):e328292. doi: 10.1136/archdischild-2024-328292 (PMC13422067; doi:10.1136/archdischild-2024-328292)
Supplement: online supplemental file 1 [file fetalneonatal-111-4-s001.pdf]

CLINICAL STUDY PROTOCOL

A Randomized Intervention, Multi-Center Study to Determine the Role of Fatty Acids in Serum in preventing Retinopathy of Prematurity

Protocol Number: MEGADONNAMEGA 16-7

Date: 180216

Sponsor and Principal Investigator: Ann Hellström MD, PhD.[1]
Tel: +46 (0)768 979196
Fax: +46 (0)31 848952

Investigator: Karin Sävman MD., PhD[1]
Tel: +46 (0)31-3432000
Boubou Hallberg MD., PhD[2]
Tel: +46 (0)8-58581354
David Ley MD., PhD[3]
Tel: +46 (0)46 17 84 40

Co-investigator: Svetlana Najm MD[1]
Dirk Wackernagel MD[2]
Mireille Vanpée MD, PhD[2]
Ingrid Pupp MD, PhD[3]

Scientific Advisers: Lois Smith MD, PhD[4]

Clinic [1]Drottning Silvias Barn och Ungdomssjukhus
[2]Astrid Lindgrens Barnsjukhus vid
Karolinska Universitetssjukhuset
[3]Lund University, Skånes Universitetssjukhus
[4]Harvard Medical School, Boston Children's Hospital

Producer of Drug: DSM Nutritional Products | 6480 Dobbin Road Columbia | Maryland 21045 | United States | T 443-542-2582 | F 410-715-6772 | Kevin Hadley, PhD.| Applied Human Evidence |
kevin.hadley@dsm.com

Study nurse Camilla Knape[1]
Ann-Charlotte Andersson[1]
Camilla Halzius[2]
Michaela Melakari[2]
Lena Legnevall[2]
Therése Kjellin[2]
Margareta Gebka[3]
Ann-Cathrine Berg[3]
Linda Nilsson[3]

Monitor: Carola Pfeiffer Mosesson
Institutionen för neurovetenskap och fysiologi, Göteborgs Universitet

This clinical study will be conducted, and essential study documentation archived, in compliance with requirements of the ICH Guidelines for Good Clinical Practice and Swedish laws

PROTOCOL SYNOPSIS

Protocol Title: A Randomized, Intervention, Multi-Center Study to Determine the Role of Fatty Acids in Serum in preventing Retinopathy of Prematurity

Protocol Number: MEGADONNAMEGA 16-7

Number of Subjects: 105 subjects receiving conventional parenteral fatty acid (Clinoleic) and enteral Arachidonic acid (AA): Docosahexaenoic acid (DHA) (Formulaid™ 2:1) supplementation and 105 subjects receiving conventional parenteral fatty acid supplementation (Clinoleic).

Subject Population: 210 premature male/female infants, born before gestational age 28 weeks + 0 days.

Dosage: All infants on parenteral nutrition from birth and as long as clinically indicated are given fatty acid supplementation (Clinoleic). In addition, children randomized to enteral AA:DHA supplementation will receive AA (100 mg/kg/d) and DHA (50mg/kg/d), from birth until 40 weeks postmenstrual age (PMA).

Study Duration: From birth to 40 postmenstrual weeks

Primary Study Objectives: A) Primary aim is to investigate whether enteral administration of AA and DHA in addition to commonly used regimes with parenteral olive based lipid emulsion (Clinoleic) reduces the sight threatening disease Retinopathy of Prematurity (ROP) compared to Clinoleic alone.

Study Objective: B. Secondary aims are to compare the two regimes with regard to;

1. postnatal serum fatty acid composition.
2. postnatal brain development, as assessed by Magnetic Resonance Imaging (MRI) Volumetric and Diffusor Tensor Imaging (DTI) at 40 weeks postmenstrual age and cognitive development at 2.0 y corrected age and 5.5 y uncorrected age.
3. neonatal glucose metabolism.
4. postnatal growth development (weight, length and head circumference).

5. frequency of neonatal morbidities such as bronchopulmonary dysplasia (BPD), cerebral intraventricular hemorrhage (IVH), patent ductus arteriosus (PDA), sepsis and necrotizing enterocolitis (NEC).

Efficacy Endpoints:

A) To compare in AA:DHA supplemented versus conventionally treated children
Serum fatty acid levels in cord blood, and at days 0, 72h, 7, 14 and every other week until postmenstrual week 29, and at postmenstrual weeks 30, 32, 34, 36 and 40, and in breast milk fatty acid levels at day 7 and postmenstrual weeks 32 and 40.
B) Incidence and severity of ROP
C) SDS score development with regard to length, weight and head circumference.
D) BPD, PDA, IVH and NEC. Brain morphology on MRI and DTI examinations.

Safety Endpoints:

Adverse events, clinical chemistry, retinal exam, physical examination, vital signs.

Table of Contents

1 INTRODUCTION 7

2 RATIONALE 10

3 STUDY OBJECTIVES 10

4 STUDY DESIGN 11

5 STUDY SUBJECTS 14

5.1 NUMBER OF SUBJECTS 14
5.2 INCLUSION CRITERIA 14
5.3 EXCLUSION CRITERIA 14

6 STUDY TREATMENT 14

6.1 INVESTIGATIONAL PRODUCT 14
6.2 STUDY DRUG ADMINISTRATION AND DOSING 15
6.3 BLINDING 16

7 STUDY CONDUCT 16

7.1 ETHICS AND REGULATORY CONSIDERATIONS 16
7.2 INDEPENDENT ETHICS COMMITTEE 16
7.3 INFORMED CONSENT/ASSENT FORM 16

8 STUDY EVALUATIONS 17

8.1 EFFICACY EVALUATIONS 17
ROP EXAMINATION 18
GROWTH 18
8.2 SAFETY EVALUATIONS 19
8.3 OTHER EVALUATIONS 20

9 SAFETY 21

9.1 RECORDING ADVERSE EVENTS 21
9.2 REPORTING SERIOUS ADVERSE EVENTS 22

10 STUDY MANAGEMENT 23

10.1 SUBJECT DISCONTINUATION 24
10.2 STUDY TERMINATION 24
10.3 DATA RECORDING 25
10.4 CASE REPORT FORMS 25
10.5 TRAINING 25
10.6 SOURCE DATA 26
10.7 QUALITY ASSURANCE 26
10.8 PROTOCOL AMENDMENTS 27
10.9 RETENTION OF STUDY RECORDS 27

11 DATA MANAGEMENT AND STATISTICAL METHODS 28

11.2 STUDY POPULATIONS 29
11.3 BACKGROUND AND DEMOGRAPHIC CHARACTERISTICS 29
11.4 ANALYSIS OF EFFICACY PARAMETERS 29
11.5 ANALYSIS OF SAFETY/TOLERABILITY 29

REFERENCES 30

APPENDIX 33

Study Administrative Structure

**Sponsor and Principle Investigator:**	**Ann Hellström MD, PhD. Professor**<br>Inst för neurovetenskap/fysiologi, Göteborg Universitet<br>Drottning Silvias barn och ungdomssjukhus/Östra<br>SE – 416 85 GÖTEBORG<br>Tel : +46 (0)31-3435774<br>Fax : +46 (0)31-3435771<br>E-mail : ann.hellstrom@medfak.gu.se
Local Investigator:	Karin Sävman MD, PhD<br>Neonatalverksamheten<br>Drottning Silvias barn och ungdomssjukhus/Östra<br>SE – 416 85 GÖTEBORG<br>Tel: +46 3432000<br>E-mail: karin.savman@pediat.gu.se
	Boubou Hallberg MD, PhD<br>Patientområdeschef/Överläkare<br>Neonatalverksamheten<br>Astrid Lindgrens Barnsjukhus vid<br>Karolinska Universitetssjukhuset<br>SE-171 76 STOCKHOLM<br>Tel: 08-58581354<br>Email: boubou.hallberg@karolinska.se
	David Ley MD, PhD. Professor<br>Neonatalverksamheten<br>Skånes Universitetssjukhus<br>SE-211 85 LUND<br>Tel: +46 (0)46 17 84 40<br>Email: david.ley@med.lu.se
**Producer of Drug:**	**DSM Nutritional Products |**<br>6480 Dobbin Road Columbia | Maryland 21045 | United States | T 443-542-2582 | F 410-715-6772 | Kevin Hadley, Ph.D.| Applied Human Evidence | kevin.hadley@dsm.com
**Laboratories:**	**Tillväxtlaboratoriet**,<br>Institutionen för kliniska vetenskaper, Sahlgrenska akademin vid Göteborgs Universitet, Vitaminvägen 21,<br>SE-416 85 GÖTEBORG<br>Email: ulrika.sjobom@gu.se

List of Abbreviations and terms

AA	Arachidonic acid
AE	Adverse Event
BPD	Bronchopulmonary dysplasia
BW	Birth weight
CNS	Central nervous system
CRF	Case Report Form
CV	Curriculum Vitae
DCF	Data clarification form
DHA	Docosahexaenoic acid
DTI	Diffusor Tensor Imaging
ECG	Electrocardiogram
EPA	Eicosapentaenoic acid
EPN	Regionala Etikprövningsnämnden i Göteborg (Ethics Committee)
GA	Gestational Age
GCP	Good Clinical Practice
GMP	Good Manufacturing Practices
GW	Gestational Week
HC	Head Circumference
ICH	International Conference on Harmonization
IVH	Intraventricular hemorrhage
LCPUFA	Long chain polyunsaturated fatty acid
MRI	Magnetic Resonance Imaging
NEC	Necrotizing enterocolitis
NICU	Neonatal Intensive Care Unit
PDA	Patent ductus arteriosus
PMA	Postmenstrual age
QA	Quality Assurance
ROP	Retinopathy of Prematurity
SAE	Serious Adverse Event
SD	Standard Deviation
SOP	Standard Operation Procedures
TMF	Trial Master File
TPN	Total parental nutrition
US	Ultrasound
VLBW	Very low birth weight

1 INTRODUCTION

Every year around 10-12 % of all infants in Europe and the US are born prematurely which results in 950000 preterm infants per year (500000 in Europe and 450000 in USA) (1). Direct complications of preterm birth account for one million deaths each year worldwide, and preterm birth is a risk factor in over 50% of all neonatal deaths. In addition, preterm birth can result in a range of long-term complications in survivors, with the frequency and severity of adverse outcomes rising with decreasing gestational age and decreasing quality of care. The annual costs, beside patient suffering and parental emotional stress, of preterm care in USA amounts to 26.2 billion USD in terms of immediate neonatal intensive care, subsequent long-term complex health care needs, as well as lost economic productivity (2).

Possible problems that may occur after a preterm birth are:

- Organ disorders (intestine, heart, lung - BPD and asthma), ears (hearing problems), eyes (ROP and visual problems)
- Feeding problems and failure to thrive
- Poor general growth
- Physical disabilities such as cerebral palsy
- Cognitive impairment
- Learning disability or behavioral problems such as attention deficit (ADD) or autism spectrum disorders. Of infants born extremely prematurely, i.e. at less than 28 weeks gestation or with extremely low birth weight (<1,000 g), 20 – 30% may show developmental disorders requiring treatment (3, 4). Impaired cognitive development and abnormal behavior may cause problems at school; in some countries the percentage of learning difficulties in the preterm population is as high as 25% (5, 6).

Much has been done to improve neonatal care e.g. target levels for oxygen saturation has been an issue for extensive discussions and clinical trials. In fact, the optimal oxygen saturation level for preterm infants has been called "a moving target," fluctuating almost as much as our patients' oxygen saturation levels. Reaching a consensus on what these levels should be is still a work in progress. Nutrient delivery is another important and central area of neonatal care which is closely associated with morbidity outcome (7, 8), and is in need of evidence-based guidelines. The project therefore aims to improve nutrient delivery to prevent or reduce the development of preterm disabilities.

There is a long tradition in neonatology to develop national and in some cases local guidelines for care in each individual Neonatal Intensive Care Unit (NICU). This has resulted in a wide range of treatment approaches and experience-based strategies. These approaches may differ between countries and hospitals and even between neonatologists at the same hospital (9). There is, for example, a fragmented approach and incomplete compliance to guidelines for nutrient delivery, one important medical parameter tightly associated with neonatal morbidities. Although over 3000 randomized controlled trials have been reported in the field of neonatology, few interventions have yet been subjected to unbiased evaluation (10).

Available nutritional fatty acid guidelines are not evidence-based and neither optimal composition nor amounts needed to meet the demands of these immature infants are known. What is known is that most infants born extremely preterm develop a large energy deficit resulting in poor neonatal growth (11). Many experience moderate hyperglycemia associated with lipid infusion (12). Hyperglycemia is a strong risk factor for preterm mortality as well as for ROP and other disorders of prematurity (9, 13). With commonly used lipid solutions, preterm new-borns experience a rapid decline in blood

fatty acid proportions of the long chain polyunsaturated fatty acids (LCPUFA) AA and DHA (14, 15, 16) , as compared to the intra-uterine situation.
In Sweden, approximately 300 infants are born extremely preterm i.e. before 28 gestational weeks yearly. With modern neonatal care, infants born as early as at 23-24 weeks of gestation, in the second trimester of gestation, have more than 50% chance of survival (17). The third trimester is a period of intense growth and differentiation of the central nervous system (CNS) of which the retina is a part, with rapid formation of synapses and dendritic spines and development of retinal photoreceptor cells. During this fetal time period AA and DHA are selectively transferred from the mother to her fetus and blood fractions of DHA increase above maternal values. AA fractions are high, twice those of the mother, from at least 24 weeks of gestation (18). After very preterm birth, the fractions of AA and DHA fall. In utero glucose, not lipid is the main source of energy and the LCPUFAs transferred during the third trimester play important structural and functional roles in membranes of the central nervous system and most other organs.

DHA, which is an omega-3 LCPUFA is derived from algae and oily fish is the predominant fatty acid of membrane phospholipids in the brain grey matter and the retina, especially its rod outer segments (19, 20). DHA is not merely a structural component of cell membranes but essential for proper function of membranes. Since the capacity to synthesize DHA is limited in humans and especially in infants, it needs to be provided in the diet (21, 22). Dietary DHA is needed for optimal functional maturation of the retina and visual cortex, it is a major component at the synaptic site, modulating the uptake and release of neurotransmitters (23, 24). Absolute accretion of DHA in the brain is greater before than after term (25) and DHA is also accumulated in adipose tissue. In addition, omega-3 LCPUFA has the potential to reduce oxidative stress, deranged glucose metabolism and inflammation.

While many studies have focused on DHA and its role in fetal and neonatal development, few studies have addressed the role of the omega-6 LCPUFA AA during fetal life and after preterm birth. Like DHA, AA is an important component of cell membranes. Altered cell membrane composition results in altered cell function. AA is abundant in the vascular endothelium and in glia where it plays different roles than DHA which is especially abundant in retinal rod outer segments and in synapses and brain grey matter. In the retina AA metabolites contribute to neurovascular coupling i.e. modulation of blood flow with neuronal activity (26). Metabolites of AA both stimulate and inhibit inflammation and angiogenesis. In addition, AA is involved in blood vessel tonus control with AA derivatives mediating both vessel relaxation and contraction (27). Low AA concentrations are associated with late onset sepsis in preterm infants (14). Improved development of very preterm infants fed twice as much AA as DHA than of infants fed equal amounts of AA and DHA was recently reported (9).

After extremely preterm birth energy expenditures increase, oral intake often takes some weeks to establish and total parenteral nutrition (TPN) is invariably required during the initial postnatal weeks. After birth lipids are the main source of energy, initially as integral part of administered TPN.
Preterm infants treated with soy and olive oil based parenteral lipid solutions (Intralipid and Clinoleic) have low levels of LCPUFAs (14) and increased supply of DHA has been recommended (15, 28). It has been demonstrated that low DHA levels are associated with compromised fetal insulin sensitivity (29) and insulin resistance in preterm

infants is common and strongly associated with neonatal morbidity. Few studies have examined associations between low AA and preterm neonatal morbidities (14).

One of the most severe morbidities affecting preterm infants is sight threatening ROP, a disorder characterized by reduced retinal vascularization followed by pathologic neovascularization which can lead to retinal detachment and blindness similar to diabetic retinopathy. ROP develops during the neonatal period and outcome is available around term age (40 postmenstrual weeks), which makes it a useful marker for short term outcome of neurovascular development in interventional studies.

In animal studies, a diet rich in omega-3 LCPUFAs reduced pathologic retinal neovascularization in oxygen induced retinopathy, through reduction of inflammatory mediators and attenuation of endothelial cell activation (30-32).
In two recent studies, the frequencies of ROP needing treatment as well as cholestasis were significantly reduced when a solution containing fish oil (SMOFlipid) was provided compared to Clinoleic in preterm infants with BW <1250 grams (33, 34). In addition, ROP frequency was reduced in infants receiving SMOFlipid as compared to those receiving Intralipid (35). No randomized controlled trial comparing conventional fatty acid administration without and with supplementation of AA and DHA from birth with regard to ROP has been published.

Oxygen supplementation concerns

Hyperoxia is a major risk factor for ROP. Most extremely preterm infants need respiratory support and receive supplemental oxygen. Optimal oxygenation has not been determined but there is some evidence that lower oxygen saturation target levels (85-89%) are associated with increased mortality compared to higher levels (91-95%) (36). Therefore, the higher target range has been implemented in some Swedish neonatal intensive care units including those which will participate in the present study. Since then the rate of severe ROP has increased substantially at Sahlgrenska University Hospital and prediction of severe ROP based on postnatal weight development has become much less efficient than with lower target limits (manuscript in preparation).
This does not necessarily mean that keeping the SpO_2 within 91-95% increases ROP and decreases the impact of other risk factors than oxygenation. At the time of the ophthalmologic examination we, at a few occasions, checked the upper oxygen saturation alarm limits and found that they varied between 84 and 98% in accordance with reports on problems with high upper alarm limits (37-40).
Manley et al. have also reported increased rates and severity of ROP after implementation of the higher SpO_2 target range (41). They speculated that a higher target range increases the tolerance by clinical staff to saturations above this range. In addition, low compliance with oxygen targeting with upper alarm limits inappropriately set too high and difficulties in maintaining saturation below the upper limit are common. Cummings et al. suggest that an upper alarm limit of 95% is reasonable (42).
Thus, to study the impact of fatty acids on ROP, meticulous oxygen control is necessary. Therefore, the target range will be 91-95% and saturation alarm limits in this study will also be 89-96%.

2 RATIONALE

Preterm Child

We have performed a randomized pilot study of 78 infants (in manuscript) comparing SMOFlipid with Clinoleic. We found no effect of SMOFlipid on ROP outcome. Infants on SMOFlipid had less loss of fraction of DHA than those on Clinoleic while fraction of eicosapentaenoic acid (EPA) increased substantially compared to cord blood and AA fell more in the SMOFlipid than in the Clinoleic group. No reduction of EPA fraction was found in the Clinoleic group. Concerns have previously been raised regarding the use of fish oil to preterm infants since both DHA and EPA may have a negative impact on proportion of AA which is thought to promote growth. No adverse effects of giving preterm infants fish-oil in SMOFlipid have been reported (1). However, raising EPA fraction and reducing AA fraction during a period of rapid growth and development appears inappropriate. We will therefore study whether a combination of AA and DHA in addition to Clinoleic compared with Clinoleic alone reduces ROP and other morbidities in extremely preterm infants and improves outcome. At present, no lipid solution for parenteral use containing significant amounts of AA and DHA is available. Instead DHA from algae and AA from fungi (Formulaid™) (DSM) are available for enteral administration. These lipids are included in preterm formula, have been given early to very preterm infants and were well tolerated (10).

The effect of different doses of DHA to preterm infants has been studied and approximately 50-80 mg/kg/day appears appropriate (43, 44). Less is known about AA needs, but in a Norwegian study 47 mg/kg/day added to breast milk resulted in less decrease in the proportion of AA in serum fatty acids (6%) than with breast milk only (24%). It was concluded that the AA dose was probably too low (43).

Choice of study drug

Most enteral formulas contain AA and DHA with ratios between 2-1:1, however no parenteral formula is today available with these fatty acids, important for development, normally transferred from mother to fetus during third trimester. Therefore, we choose Formulaid™ with a 2:1 AA:DHA ratio to be administered orally from birth to 40 weeks PMA.

3 STUDY OBJECTIVES

A. Primary aim is to investigate whether enteral administration of AA and DHA in addition to commonly used regimes with parenteral olive based lipid emulsion (Clinoleic) compared to Clinoleic alone prevents the sight threatening disease Retinopathy of Prematurity (ROP).

B. Secondary aims are to investigate;

1. postnatal serum fatty acid composition in preterm infants with and without AA:DHA supplementation.
2. postnatal brain development, as assessed by Magnetic Resonance Imaging (MRI) Volumetric and Diffusor Tensor Imaging (DTI) at 40 weeks postmenstrual age and motor and cognitive development at 2 years corrected age and 5.5 uncorrected age..
3. neonatal glucose metabolism.
4. postnatal growth development (weight, length and head circumference).

5. frequency of neonatal morbidities such as bronchopulmonary dysplasia (BPD), cerebral intraventricular hemorrhage (IVH), patent ductus arteriosus (PDA), sepsis and necrotizing enterocolitis (NEC).
6. postnatal body composition at 40 weeks by Pea-Pod

At 2 years corrected age growth measurements, ophthalmologic examination as well as neurological and cognitive evaluation will be performed. At 5.5 y uncorrected age growth measurements as well as cognitive testing and behavioral questionnaire will be performed. Health economic aspects with regard to morbidities and quality of life outcomes in the two groups will be investigated. Extensive ophthalmologic examination, including visual perception and morphologic and functional examination of the retina, will be performed at 6.5 years.

4 STUDY DESIGN

The study is a Randomized Intervention, Multi-Center Study to Determine the Role of Fatty Acids in Serum and Breast Milk in preventing Retinopathy of Prematurity Subjects who meet all inclusion and none of the exclusion criteria will be enrolled into the study. Upon entry into the study, subjects will be randomized and given a unique subject number.
A randomized intervention study of 105+105 (number based on power analysis regarding up to date ROP frequency, see 5.1 and 11.1) infants without major malformations born with a gestational age less than 28 weeks + 0 days will be performed.
A. Conventional parenteral fatty acid treatment with Clinoleic

B. **Enteral supplement of AA (100mg/kg/day) and DHA (50 mg/kg/day) from birth to 40 weeks postmenstrual age in addition to conventional parenteral fatty acid treatment with Clinoleic.**

Enteral supplementation with AA:DHA will start at second enteral feeding after birth and continue once daily to postmenstrual week 40 + 0. The supplementation will be delivered prior to feeding (0.1-1ml, according to dosing scheme, Appendix A). If the infant does not tolerate any enteral feeding, the supplement will be given as long as gastric retention is administered.
The intervention group will receive a daily dose of 100 mg AA/kg/day and 50 mg DHA/kg/day (Formulaid™ 2:1 DSM). Dose adjustment will be performed after the infant has regained its birthweight and weight gain results in an increase of 0.1 ml or more (see Appendix A).

Formulaid™ is produced from a blend of algal, fungal, and high oleic sunflower oils, and contains AA and DHA (triglyceride form) in a ratio of 2:1. Approximate concentrations are 265 mg/g and 135 mg/g. The study oil will be dispensed 1ml purple syringes long time stored at -80 C. Syringes are then provided to the neonatal ward where they can be stored for up to three months in +4 - +8 °C. .

As we will have clinical record forms (CRF's) online the randomization is given in the CRF when the patient's parents have agreed to participate in the study.

Randomization will be as follows; GA ≤ 24 weeks +6 days, n= 84, GA 25 weeks +0 days to ≤ 26 weeks +6 days, n= 84 and GA 27 weeks +0 days to ≤ 27 weeks +6 days,

n=42 (in order to receive equal number of infants with conventional treatment and infants treated with AA:DHA supplementation in relation to morbidity outcome. In order to adjust for center variability each center (n=3) will recruit as follows; GA ≤ 24 weeks +6 days, n= 14 (conventional) +14 (treated), GA 25 weeks+0 days to ≤ 26 weeks +6 days, n= 14 (conventional) +14 (treated) and GA 27 weeks +0 days to ≤ 27 weeks +6 days, n=7 (conventional) +7 (treated).

The treating nurse/doctor will receive the randomization on-line. The examiners (ophthalmologists, radiologists and psychologists) will be blinded for fatty acid treatment regime. The duration of parenteral nutrition and the amount of fatty acids administered will be according to clinical routines.

Thus there is one group of infants (n=105) that will receive AA and DHA supplementation from birth to 40 postmenstrual weeks.

Data collection
After we have received informed consent from the parents/guardians, blood samples from the infant will be taken according to present clinical practice. If possible 2ml cord blood and 0.6 ml blood at days 0, 72h, 7, 14 and from that time point every second week until postmenstrual week 29, and thereafter at 30, 32, 34, 36 and 40 weeks postmenstrual age will be taken.
Breast milk samples will be taken day 7 and at PMA of 32 and 40 weeks. Length, weight and head circumference are measured weekly.
Screening for ROP will be performed, at least once a week, according to clinical routines using a specific protocol.
We intend to analyze the content of phospholipids which can be done on small amounts of blood, is relatively insensitive to short term fluctuations in intake and mirror the composition of many membranes in the body. The analyses will be made using gas-liquid chromatography. The method has a coefficient of variability of 1-3% for the fatty acids concerned.

Mega Donna Mega Site: Schedule Study nr:___________________

	day 0	day 1	day 2	hour 72	day 4	day 5	day 6	day 7	day 14	W 3	W 4	W 5	W 6	w 7	PMA 30+0 +- 24h	PMA 31+0 +- 24h	PMA 32+0 +- 24h	PMA 33+0 +- 24h	PMA 34+0 +- 24h	PMA 35+0 +- 24h	PMA 36+0 +- 24h	PMA 37+0 +- 24h	PMA 38+0 +- 24h	PMA 39+0 +- 24h	PMA 40+0 +- 24h
**Datum**																									
**Gestational age (weeks+days)**																									
Patient info/consent within 36 h	x																								
Physical exam	x																								
Weight+ Length + Head circum-ference ± 3d from sampling	x							x	x	x	x	x	x	x	x	x	x	x	x	x	x	x	x	x	x
Registration of HR+MABP every second hour up to day 7 (excel)	x	x	x	x	x	x	x	x																	
Study sample cord blood (2 ml). 0h, 72h, d7, d14 (+- 6 h) every other week until PMA 29w and thereafter at 30, 32, 34, 36, and 40 weeks PMA (0,6 ml), (+- 24 h).	x			X				x	x		x		x		x		x		x		x				x
Blood gas	x																								
Samples of ventricular & tracheal as-pirate		X																							
Chemlab Bilirubin total and conjugated								x	x								x								x
Breast milk samples d7, w32 (same day as study sample), w40								x									x								x
Ultrasound brain day 1, 72 h, day 7, once between day 21-35 and once between w 32-40 Pea-Pod Lund och GBG		x		x				x		X d21-35							X w32-40								Pea-pod X
Ultrasound heart before Pedea treatment, if not treated at d 7				x				x																	
ROP exam with Retcam + SWEDROP																x		x		x		x		x	
Lung provocation test, +/- 3 days																					x				
MRI																									x
Questionnaire day 7, week 40								x																	x
Questionnaire about Retcam																	x								
Bakterieprover day 3-5, 14, 28, week 34 senast				X					X		X								X						

5 STUDY SUBJECTS

5.1 Number of Subjects

For efficacy evaluations, 80 subjects need to be included in each interventional arm for statistical considerations. However, to compensate for protocol violations, mortality and withdrawals up to 105 subjects in each arm will be included (see 11.1 Termination of Sample Size and Statistical Methods on page 26).
After the first 30 subjects have been treated and evaluated there will be a safety check to confirm that the safety profile is acceptable and that the assumption of reduction in ROP incidence is reasonable.

5.2 Inclusion Criteria

Subjects must meet all the following inclusion criteria to be permitted into this study:

1. Signed informed consent from parents/guardians;
2. Subject must be born before 28 weeks of gestation

5.3 Exclusion Criteria

Subjects presenting with any of the following will be excluded from the study:

1. Detectable clinical gross malformation;
2. Known or suspected chromosomal abnormality, genetic disorder, or syndrome, according to the investigator’s opinion;
3. Clinically significant neuropathy, nephropathy, retinopathy, or other micro- or macrovascular disease requiring treatment, according to the investigator’s opinion;
4. Any other condition or therapy that, in the investigator’s opinion, may pose a risk to the subject or interfere with the subject’s ability to be compliant with this protocol or interfere with interpretation of results.

6 STUDY TREATMENT

6.1 Investigational Product

DSM has received marketing authorization for Formulaid™ for enteral fortification of preterm formulas.

DSM will supply Formulaid™ 2:1 for the study. The local hospital at each study site will be responsible for storing and dispensing all supplies.

The formulation will be stored at -80 C, until patient is included in the study
When a child is included in the study and randomized to receive Formulaid ™ the department receives the number of feeding syringes that the baby needs during the study period. The prefilled feeding syringes can be stored in the refrigerator.

6.2 Study Drug Administration and Dosing

Regarding fatty acid supplementation to preterm infants the today's regime at all neonatal intensive care units in Sweden is to prepare a mixture of Vitalipid infant and Soluvit with Clinoleic. In addition, according to today's clinical praxis and pharmacy regimes the preparation of Clinoleic, Vitalipid infant and Soluvit lasts for seven days if stored at +4 to +6 and prepared by the local pharmacy. Infants randomizes to Formulaid™ will additional to Clinoleic receive 0,1-1ml daily administered ***enteral as an oral drug***. Start of administration will start on the first day of life (within 24 hours) preferably after the first enteral feeding in association with the second feeding. If the infant of some clinical reason cannot receive Formulaid™ within the first 24 hours this should be reported as an AE.
Formulaid™ (100 mL) will be thawed overnight in refrigerator and then left to reach room temperature before being allocated to oral syringes.
One mL oral syringes with cap will be filled with Formulaid™ and then stored in -80 degrees C at each responsible clinic.
At inclusion of an infant the number of study ID marked syringes needed throughout the study period will be allocated. These will be stored in refrigerator at the ward or at home.

Preterm Child – Treated

All infants will receive parenteral and enteral nutrition according to clinical practice and routines.
The group of infants randomized to treatment with enteral Formulaid™ will receive AA and DHA with a quotient that is 2:1. The time period for the enteral supplementation will start during the first day of life until 40 weeks postmenstrual age. The dose given will be 100 mg AA/kg/day and 50 mg DHA/kg/day, i.e. with volumes ranging from 0.1 ml to 1 ml/day (see Appendix A for details in dosing).

Preterm Child - Conventional fatty acid supplementation

A preterm child that is randomized to conventional treatment receives Clinoleic (with Vitalipid infant and Soluvit supplementation) according to regular clinical practice and routines as described below.
For all preterm infants the parenteral nutrition with glucose, amino acids and lipids is introduced within the first 24 hours of life. The infusion of lipid emulsion (Clinoleic 20%) is gradually increased during the first days of life to a maximum of 3,5g/kg/24 h at day 3 to 5 of life. Consequently, a preterm infant with a birth weight of 1 kg will receive a maximum of 15 ml lipid emulsion/24 h (3 g/kg) which, according to Brans et al (45) is within acceptable levels (i.e 2-3 g/kg/day) for Very low birth weight (VLBW) infants. Enteral feeding with human breast milk is introduced from the first day of life. According to our clinical practice we start with 1-5 ml/kg every third hour depending of maturity and birth weight. The amount of breast milk is slowly and carefully gradually increased during the first 7 to 10 days of life until the infant is fully enteral fed. With increasing enteral amounts the parenteral dose of lipid emulsion is decreased. Some of the most immature infants may have problems to tolerate increased enteral feeding leading to a longer period of parenteral feeding in low

doses. In conclusion, feeding VLBW infants according to clinical practice and routine are a regiment with *partial* parenteral nutrition combined with enteral nutrition.
If an infant does not tolerate any enteral feeding (for example gut problems as NEC or during any kind of surgery) give Formulaid™ before giving back the retention and flush the probe with little air afterwards. It is up to the responsible clinician to determine if Formulaid™ can be administered to the individual child.

6.3 Blinding

This is a randomized study with blinded ophthalmologic assessment of ROP stage. Retinal examination will be performed approximately once weekly starting at four to five weeks of age according to a standardized protocol and to clinical screening praxis. The evaluation of ROP occurs independently from the study and pediatric ophthalmologists will be unaware of which infants are participating in the study.

7 STUDY CONDUCT

7.1 Ethics and Regulatory Considerations

This study will be conducted in accordance with current Good Clinical Practices (GCPs) and International Conference on Harmonization (ICH) recommendations, as well as all applicable local, state, and federal regulations and guidelines regarding the conduct of clinical trials.

7.2 Independent Ethics Committee

The protocol, informed consent form, and other written subject information must be submitted to the “Regionala Etikprövningsnämnden i Göteborg” (EPN) www.epn.se and their written unconditional approval must be obtained prior to commencement of the study.
Verification of unconditional approval from EPN of the protocol and the approved informed consent form will be forwarded to DSM, the manufacturer of the study medication, prior to shipment of study medication supplies to the site.

7.3 Informed Consent/Assent Form

Informed consent by the parents/guardians for each subject will be obtained before initiating any study procedures. Both parents/guardians will have to sign the informed consent. Informed consent should be obtained as soon as possible after birth and no later than 36 hours after birth. One copy of the signed and personally dated informed consent must be given to each parent/guardian and one signed and personally dated copy must be retained in the investigator’s trial records. The “Declaration of Helsinki” recommends that consent be obtained from each potential subject or parents/guardians in biomedical research trials after the physician has explained to the individual the purpose, methods, anticipated benefits, and potential hazards of the trial and discomfort it may entail.

Potential subject’s parents/guardians should also be informed of their right not to participate or to withdraw from the study at any time. If the individual is in a dependent

relationship to the physician or gives consent under duress, an independent physician should obtain the informed consent. If the individual is legally incompetent (i.e., a minor or mentally incompetent), informed consent must be obtained from the parents, legal guardians, or legal representative in accordance with the law in Sweden. See the "Declaration of Helsinki".

If a protocol amendment substantially alters the study design or there is an increase of a potential risk to the subject:

- the informed consent form or subject information sheet must be revised and submitted to EPN for review and approval; and
- the approved revised form must be signed by parents/guardians to subject currently enrolled in the study; or
- the new form must be used to obtain consent from new parents/guardians prior to enrollment into the study.

7.4 Subject Data Protection

The Investigator is responsible for keeping a list of all subjects (who have been allocated subject numbers) including subject numbers, full names (of parents/guardians and child, if applicable) and parents addresses.

The parents/guardians should also be informed in writing and agree to the possibility of audits and /or monitoring by authorized representatives of the Sponsor, the manufacturer of the study drug, and/or regulatory authorities in which case a review of those parts of the laboratory records relevant to the study may be required.

The parents/guardians should be informed in writing and agree to that the results will be stored and analyzed in a computer, maintaining confidentiality in accordance with the Swedish Personal Data Ordinance (1998:1191). "Personuppgiftslagen" (PUL SFS 1998:204, SFS 1998:1191)

7.5 Biobank

Samples taken in this study will be stored in a biobank according to the National Swedish Board of Health and Welfare in accordance with the Biobanks in Medical Care Act (2002:297) The biobank is registered at the national board of health and welfare in Sweden (Socialstyrelsen). The parents/guardians will be informed about this.

8 STUDY EVALUATIONS

8.1 Efficacy evaluations

All values obtained from efficacy evaluations should be recorded onto the electronic case record form (eCRF). Serum samples will be taken simultaneously with clinical blood sampling.

Fatty acid analyses

The analyses of fatty acids will be performed at the University of Gothenburg, where the method has been developed and validated on the limited volumes available in pre-term infants. Total lipids of serum will be extracted according to Folch et al. (46). Serum lipids will be fractionated on a single SEP-PAK aminopropyl cartridge (Waters Corp., Massachusetts, USA) and phospholipids eluted with methanol after washing with chloroform:isopropanol 2:1 and 2% HAc in ether. The phospholipid fraction will be transmethylated in methanolic-HCL-3N at 80°C for 4 hours. The method has been described earlier (47). The fatty acid methyl esters will be separated by capillary gas-liquid chromatography in an Agilent gas chromatograph, *supplied by* Department of Biological and Environmental Sciences, University of Gothenburg. The separation will be recorded with Agilent GC-MS Chem Station software and the data analyzed using Agilent MassHunter software. The fatty acid methyl esters will be identified by comparison with retention times and mass spectra of pure reference substances (Sigma Aldrich Sweden AB, Stockholm, Sweden and Larodan AB, Solna, Sweden).

ROP examination

The retinal examination will be performed once weekly starting at five to six weeks of age but not earlier than at postmenstrual age of 31 weeks, according to a standardized protocol and to clinical screening praxis. The ophthalmologic assessment will be performed with strict criteria according to general Swedish Guidelines issued by the Swedish Ophthalmological Society: The Guidelines are available at following link: www.swedeye.org/SOTA/rop/SOTA-ROP_2006.pdf.

Growth

Length, weight and head circumference will be registered weekly from birth until 40 weeks postmenstrual age and at 2.0 y corrected age and at 5,5 y uncorrected age.

Body Composition (Lund and Gothenburg)

Peapod will be performed at postmenstrual age at 40 weeks followed by DXA at 2.0 y corrected age and at 5,5 y uncorrected age

Brain/Neurologic development

Cranial ultrasound will be performed postnatal days 3 and 7 and once between days 21-35 and once between postnatal weeks 32-40. MRI and DTI of the brain will be performed at 40 weeks PMA.

At 2.0 y corrected age a clinical examination including neurologic evaluation (neurologist) cognitive evaluation with BaileyIII-test (psychologist) will be performed and at 2.5 years an ophthalmologic examination will be performed.

At 5.5 years a clinical examination including neurologic evaluation (neurologist) cognitive evaluation with WPPSI-IV and behavioral questionnaire SDQ, short visuo-motor test will be performed. Extensive ophthalmologic examination, including visual

perception and morphologic and functional examination of the retina, will be performed at 6.5 years.

Morbidities BPD, IVH, PDA, NEC will be assessed at 40 weeks PMA according to the SNQ register.

8.2 Safety Evaluations

All values obtained from safety evaluations should be recorded onto the CRF. Safety evaluations consist of the following:

- **AE reporting**: include SAEs and AEs related to the study drug shall be recorded starting from receiving informed consent until the final study examination / sampling. AE reporting is further outlined in section 9, Safety.
- **Physical examination:** A complete physical examination will be performed in all subjects at birth according to standard clinical routines.
- **Vital signs:** Heart rate, blood pressure, pO_2 and breath frequency will be monitored at birth and recorded in the eCRF in VIEDOC. Followed by registration of HR and MABP in excel every second hour up to day 7 PMA. Subjects are connected with continuous surveillance of heart beat, breath frequency and pO_2. An alarm will notify the personnel and if any true divergences have occurred, these will be recorded in the patient record. Children with artery catheter are always under continuous intra artery blood pressure control with alarm for any irregular episodes
- Samples of ventricular & tracheal aspirate: At postnatal day 1 gastric fluid will be taken by the insertion of a feeding tube early after birth and analysed for composition e.g.inflammatory and oxidative biomarkers and fatty acids. Tracheal aspirate will be obtained during routine endotracheal suctioning daily in infants treated on ventilator and analysed for composition e.g. inflammatory and oxidative biomarkers and fatty acid composition.
- Retinal exam: Retinal exam will be performed according to clinical screening protocol. Any deviations from normal will be described in the CRF. RetCam images shall be taken if doable at each screening examination and has at least to be taken prior to treatment of severe ROP.
- Cranial/Cerebral ultrasound will be performed in all subjects by an experienced neonatologist or a pediatric radiologist as clinical routine. Any deviations from normal will be described in the CRF.

- **Cardiac Ultrasound - PDA** will be performed in all subjects by an experienced neonatologist or a pediatric cardiologist as clinical routine prior to PDA treatment. If the baby has not been treated for PDA, ultrasound will be performed day 3 and day 7.
- The following variables will be registered in the CRF;

1. Width of ductus: xx mm
2. Dominating direction of the shunt through DA: left-right/equilibrium/right-left
3. Speed of DA shunt: yy m/sec

4. La/Ao quotient:
5. Reversed diastolic flow in post-ductorial aorta: yes / no

- **Magnetic resonance imaging of the brain** will be performed in all subjects by a pediatric radiologist as clinical routine. Any deviations from normal will be described in the CRF.
- **Laboratory evaluations**: Laboratory assessments will be performed according to standard clinical routines. Blood samples for fatty acid analyses; 2 ml from cord blood and 0.6 ml from the child will be taken according to present clinical practice at 0h, 72h, d7, d14, every other week until PMA 29w, and then at PMA 30w, 32w, 34w, 36w, and 40 weeks. Breast milk samples are taken day 7 and postmenstrual weeks 32 and 40. Length, weight and head circumference are measured weekly to a PMA of 40 weeks.
  Analyses of triglycerides cannot be taken more frequently then described in the protocol as the small circulating blood volume of these very preterm infants will not allow more sampling than what is already specified in the protocol.
  It is extremely rare in our neonatal intensive care unit that a WLBW infant is planned to receive more than 3 g fat/kg/24 h intravenously, but if it should happen the infant is always very thoroughly followed by clinical checkups with daily measurements of plasma triglycerides and blood gases (as a safety control of metabolic state).

  Laboratory evaluations consist of:

  Chemistry: Total and conjugated bilirubin at days 7, 14 and at postnatal weeks 32 and 40.

  Plasma glucose: Blood for measurement of p-glucose is obtained from routine blood gas measurements (taken prior to feeding) and will be recorded in the CRF throughout the study.

8.3 Other Evaluations

- **Maternal and perinatal history:** Maternal and perinatal history will be recorded as soon as possible after birth after questioning the subject´s parents and/or recorded from the mother’s patient record for the pregnancy.

- **Maternal dietary habits**

  A questionnaire regarding maternal dietary habits will be performed after birth at day 7 to retrospectively register eating habits during pregnancy and at 40 weeks PMA to register dietary habits during postnatal care.

- **“Patient/parent” involvement**

  A validated questionnaire "Parent's concerns about participating in this clinical trial" on preterm infants will be used to collect data at the beginning, and at the end of the clinical trial period. In addition, a parent’s focus group will form and meet twice a year to discuss a family-centered approach to clinical research. Research questions include: (1) The perceived challenges of being involved in a clinical trial; (2) The perceived ad-

vantages of being involved in a clinical trial; and (3) The experiences of patients at different points in the clinical trial to improve interaction and joint decision making in the present study. We will start a "patientråd" in which a number of preterm patient associations (including EFCNI-www.efcni.org) are invited to one meeting/year during which improvements in care and clinical studies in preterm infants will be discussed.

- **Care at Retcam examination (Sthlm ,Lund & Gothenburg)**

  A questionnaire regarding parent's experience of Retcam examination and the baby's wellbeing during and after the examination, will be performed at the first Retcam examination. We also want to correlate the answers to the baby´s medical records. Different nursing care techniques will be compared and correlated to the baby's wellbeing to find the most optimal care.

- **Examination of the intestinal flora**

  When the child is 3-5 days, 14 days, 28 days, and at discharge from the neonatal department, but not later than PMA 34 weeks, feces will be collected. At the same time four sterile cotton swabs will be rotated in the oral cavity of the infant. The feces and the cotton swabs will be sent for microbiological analyzes to study the early pattern of the normal intestinal. This is done to map the child´s early normal bacterial flora and if the flora is affected by the intake of Formulaid™.

9 SAFETY

9.1 Recording Adverse Events

Adverse events related to study drug must be recorded starting from the time of informed consent until the final study examination / sampling day. Any medical condition present at the initial study day (birth day), which remains unchanged or improves, should not be recorded as an adverse event at subsequent examination / sampling days. However, if there is **deterioration** of a medical condition that was present at the initial study day (birth, day 0), this should be considered a **new** adverse event and reported. This information is collected by examining the subject. Ongoing adverse events at the final study day should be followed until the event is resolved or remains stable.

Clinically significant changes (abnormalities), in the judgment of the investigator, in physical examination from the baseline exam will be recorded as an adverse event.

The following information must be collected and recorded for each AE:
- AE term (diagnosis)
- Action taken regarding AE
- AE outcome (resolved, ongoing, death, or lost to follow-up)
- AE causality (not related, possibly related, related)

The investigator should determine study drug relationship for each adverse event. AEs occurring prior to administration of the study drug will be considered as not related to

the study drug. The Investigator will be using the following explanations for assessment of causality:

Not related

- The event is clearly related to other factors such as the subject's clinical state, therapeutic interventions, or concomitant drugs administered to the subject.

Possibly Related

- The event follows a reasonable temporal sequence from the time of drug administration,
- And/or follows a known response pattern to the trial drug,
- **But** could have been produced by other factors such as the subject's clinical state, therapeutic interventions, or concomitant drugs administered to the subject.

Related

- The event follows a reasonable temporal sequence from the time of drug administration,
- **And** follows a known response pattern to the study drug,
- **And** cannot be reasonably explained by other factors such as the subject's clinical state, therapeutic interventions, or concomitant drugs administered to the subject,
- **And** either occur immediately following trial drug administration, **or** improves on stopping the drug, **or** reappears on repeat exposure, **or** there is a positive reaction at the application site.

It is up to the discretion of the Investigator, in the event of an AE or SAE, to temporarily discontinue study medication or to discontinue the subject from the study. The end of study examinations / samplings must be performed if the subject is discontinued from the study.

9.2 Reporting Serious Adverse Events

A serious adverse event (SAE) related to the study drug is defined as any adverse drug experience occurring at any dose that results in any of the following outcomes:

- Results in death,
- Is life-threatening (NOTE: The term "life-threatening" in the definition of "serious" refers to an event in which the subject was at risk of death at the time of the event; it does not refer to an event which hypothetically might have caused death if it was more severe).
- Results in inpatient hospitalization or prolongation of existing hospitalization,
- Results in a persistent or significant disability/incapacity, or
- Results in a congenital anomaly/birth defect.

Important medical events that may not result in death, be life-threatening, or require hospitalization may be considered a serious adverse drug experience when, based upon

appropriate medical judgment, the event may jeopardize the subject and may require medical or surgical intervention to prevent one of the outcomes listed in this definition.

Any SAE occurring during the study (from receiving informed consent until end of study) or within 30 days after study completion will be reported to the Sponsor within 24 hours of knowledge of the event.

In the event of an SAE, the SAE/DSM form available in the electronic Trial Master File (TMF) is to be completed and reported to UBC Geneva by fax (+800 24 25 26 27 or +41 22 596 44 46) within 24 hours of learning about the event regardless of whether all information is known. An e-mail will also be sent to John.Cianflone@dsm.com to inform about the SAE.

Initial SAE reporting can be done by telephone with written reports to follow by fax within 24 hours.

All SAEs must be followed until resolution (subject has returned to baseline status of health), or until stabilization (the investigator does not expect any further improvement or worsening of the reported event).

Sponsor Safety Contact Information

Contact:	Ann Hellström
Title:	MD Professor
Address:	The Queen Silvia Children's Hospital<br>Göteborg University/Östra<br>SE – 416 85 GÖTEBORG
24 hour line:	+46 (0)768 979196
Office:	+46 (0)31 3435774
Fax:	+46 (0)31 3435771

Safety Contact Information for DSM:

Contact:	UBC Geneva
E-mail:	EUsafety@ubc.com
Fax:	+800 24 25 26 27
Tel:	+41 22 596 44 46

10 STUDY MANAGEMENT

A total of 210 subjects, according to the following schedule:

- First subject enrolled: [20160915]
- [35] subjects enrolled: [20170501]
- [70] subjects enrolled: [20170715]
- [140] subjects enrolled: [20171215]
- [210] subjects enrolled: [20180501]

- Last subject completed: [20180901]

10.1 Subject Discontinuation

A discontinuation occurs when an enrolled subject ceases participation in the study, regardless of the circumstances, prior to completion of the protocol.

The investigator has the right to remove a subject at any time if it is in the best medical interest of the subject.

Subjects will be discontinued from treatment prior to completion for any of the following reasons:

- Consent withdrawn / the parents/guardians wish to discontinue study treatment
- AE (clinical events or laboratory values) that contraindicate continuing the study.
- Best interest of the patient, as judged by the Investigator
- Protocol violation
- Administrative Decision
- Other, by Investigator specified reason

Subjects withdrawn from the study for a SAE should be followed until the SAE has resolved. Appropriate supportive and/or definitive therapy should be administered as required.

The investigator must determine the primary reason for discontinuation. The reason for a subject discontinuing from the study will be recorded on the case report form. Withdrawal due to an adverse event should be distinguished from withdrawal due to other reasons, according to the definition of an adverse event noted earlier. A discontinuation must be reported immediately to UBC Geneva by fax (+800 24 25 26 27 or +41 22 596 44 46) if it is due to a serious adverse event.

The **End of Study** examination / sampling must be performed at the time of the study discontinuation. The investigator will record the reason for study discontinuation, provide or arrange for the appropriate follow-up (if required) for such subject, and document the course of the subject’s condition.

10.2 Study Termination

Termination of the study before all subjects have been enrolled can occur for any of the following reasons:

- it was determined that the risk level associated with the experimental drug was significant and warranted termination of the study;
- the sponsor terminated the study for any reason, at any time, by written notice of intended termination;
- the principal investigator, EPN terminated participation of that clinical site in the study by written notice;

- any other clause in the individual site Clinical Study Agreement was not met

10.3 Data Recording

Source documents are original documents, data, and records from which the subject's case report form (CRF) data are obtained. These may include but are not limited to hospital records, clinical and office charts, laboratory and pharmacy records, diaries, microfiches, radiographs, and correspondence. All original source documents supporting entries on CRFs must be maintained and be readily available.

The investigator will record all data with respect to the study in the subject's CRFs. This includes but is not limited to study procedures, laboratory data, safety-related data and drug accountability.

The investigator will sign and date the indicated places on the CRFs. These signatures will signify that the investigator inspected or reviewed the data on the CRF and on the data queries and that he/she agrees with the content.

All corrections on a CRF and on source documents must be made in a way that does not obscure the original entry. The correct data must be inserted, dated and initialed by study center personnel. If the reason for the change is not obvious, an explanation should be provided.

10.4 Case Report Forms

The Investigator will complete Case Report Forms for all subjects. These are to be completed in English. If a test/assessment is not done and will not be available, indicate this by writing "N/D" (Not Done) in the respective answer field in the CRF. If the question is irrelevant (e.g. is not applicable) indicate this by writing "N/A" (Not Applicable) in the respective answer field.

Corrections of data can only be made by crossing out the incorrect data and writing the correct data next to those crossed out (e.g. ~~352~~ 325). Erasure by any method is not allowed. Any changes in the CRF by the Investigator or his/her delegate must be signed with initials, dated and explained (if necessary). If corrections are made by the Investigator's authorized staff after the date of the Investigator's signature on the CRF, the CRF must be signed and dated again by the Investigator. Corrections necessary after the CRFs have been removed from the Investigator's site must be documented on a Data Clarification Form (DCF).

Original source documents, case report forms, and other study documentation will be maintained at the study site as specified.

Completed original Case Report Forms are the property of the Sponsor.

10.5 Training

The Investigator will ensure that appropriate Good clinical practice training relevant to the study is given to the medical, nursing and other staff involved. Any information of

relevance to the performance of this study is to be forwarded to the co-investigator and other staff involved.

All investigators signing the protocol and key personnel should provide signed and dated Curriculum Vitae (CV) originals to be filed by the Sponsor. The CV should include name, title, occupation, education, research experience and present and former positions. A staff signature list including delegation responsibilities is required and will be continuously up-dated.

10.6 Source Data

Source data will be collected in the "Source Data File". The Source Data File should contain raw data from examinations/samplings/laboratory results required to verify the data entered into each patient's CRF.

The following data will be recorded directly on the electronic CRFs and will be considered source data: gender, age, length, weight, HC, medical history, physical examination and retinal scan.

The hospital records should clearly indicate at least:

- that the patient participated in the study (by patient identification and study identification)
- when the written informed consent was obtained,
- all examinations/samplings/laboratory results of importance for the patient's clinical care and
- serious adverse events.

10.7 Quality Assurance

The data will be entered into a database, where internal review and programmed computer checks will be used to identify selected protocol violations and data errors. If necessary, requests for clarifications or corrections will be sent to the investigator.

The investigator agrees to monitoring of the study by a Sponsor representative and that Regulatory Authorities will have the right from time to time both during and after the course of this trial to inspect the study and review pertinent medical records relating to this clinical trial.

Before, during and after the study, the monitor will have regular contacts with the clinic including visits to confirm that facilities remain acceptable, that the investigational team is adhering to the protocol, that data are being accurately recorded in the CRF and to provide information and support to the Investigator. Monitoring of the

study will be carried out by clinical research manager Carola Pfeiffer Mosesson, Institute of Neuroscience and Physiology, University of Gothenburg according to their Standard Operating Procedures (SOP).

A statement will be obtained from each subject's parents/guardians participating in the trial permitting the release of the subject's medical records as necessary for monitoring or inspection by authorized personnel for the Sponsor and Regulatory Authorities.

The investigator is responsible for maintaining a comprehensive and centralized filing system of all study-related (essential) documentation, suitable for inspection at any time by representatives of the Sponsor and Regulatory Authorities.

10.8 Protocol Amendments

All revisions to the protocol should be reviewed and approved by the Sponsor prior to submission of the amendment to the regulatory authorities. If the revision is an Administrative Change, the investigator should submit it to the EPN for their information. If the revision is an Amendment to the protocol, the Investigator must sign it to verify he/she has read and understands the change. The investigator must submit the Amendment to the EPN for review and approval prior to implementation.

If an amendment substantially alters the study design or increases the potential risk to the subject:

- the informed consent form or subject information sheet must be revised and submitted to the EPN for review and approval; and
- the approved revised form must be signed by both parents/guardians currently enrolled in the study; or
- the new form must be used to obtain consent from new parents/guardians prior to their enrollment into the study.

10.9 Retention of Study Records

ICH-GCP guidelines require the medical records and notes, etc., should be clearly marked and permit easy identification of participation by an individual in the trial.

The files should be archived by the investigator at least 10 years after the study is finished.

The investigator is to record all data with respect to protocol procedures, drug administration, laboratory data, safety data, and efficacy data on the CRFs. Essential documents should be retained until at least 2 years after the last approval of a marketing application in an ICH region and until there are no pending or contemplated marketing

applications in an ICH region or at least 2 years have elapsed since the formal discontinuation of clinical development of the investigational product. The investigator should store the study records in a secure location.

11 DATA MANAGEMENT AND STATISTICAL METHODS

The investigator will record all data with respect to the study in the subject's CRFs. This includes, but is not limited to, study procedures, laboratory data and safety-related data.

All corrections on a CRF and on source documents must be made in a way that does not obscure the original entry. The correct data must be inserted, dated and initialed by study center personnel. If the change is not obvious, an explanation should be provided.

Completed CRFs for this study will be forwarded to the sponsor for editing, construction of a quality-assured database, and analysis of the data.

Descriptive and analytical statistics will be used.

11.1 Determination of Sample Size and Statistical Methods

Primary endpoint

The mean incidence of any ROP in an age-matched population (22 weeks + 0 days to 27 weeks + 6 days of GA at birth) in Sweden during the years 2008 – 2012 is calculated to 42%. Assuming an alpha of 5%, a power of 80% and a 50% reduction in incidence of any ROP, i.e. from 42% to 22%, a sample size of 80 subjects per treatment group is required. The reduction in any ROP with AA:DHA supplementation is based on efficacy in previous publications (33).
A total of 105+105 subjects will be included to compensate for protocol violations (experience gained in pilot study) and drop-outs (e.g. a perinatal death rate of approximately 15% at these gestational ages).
After the first 10 subjects have been treated, we will perform pharmacokinetic analyses with respect to fatty acid concentrations in serum.
After the first 30 subjects have been treated and evaluated a safety data committee will perform an evaluation to confirm that the safety profile is acceptable and that the assumption of a reduction in ROP incidence is reasonable.

Secondary endpoints

Important secondary endpoints are:

- Body weight
- Length
- Head circumference

These will be modelled by a linear mixed effects model. Subject will be used as a random factor, and *GA* and *treatment* will be used as fixed factors.

11.2 Study Populations

All available subjects will be used in the data summaries and the listings of subject data. If a subject is regarded as non-evaluable this subject may be listed separately and not included in the summary statistics.

11.3 Background and Demographic Characteristics

The background and demographic variables will be displayed in per subject listings. The quantitative variables will also be summarized by means, standard deviations and medians. The qualitative variables will be displayed in frequency tables.

11.4 Analysis of Efficacy Parameters

Fatty acid analysis
Serum concentrations of fatty acids will be reported for the child and mother and displayed in per subject listings and graphs. The efficacy parameters will be displayed in per subject listings and summarized by means, standard deviations and median values.

ROP evaluation
The ophthalmologic assessment will be performed with strict criteria according to general Swedish Guidelines issued by the Swedish Ophthalmological Society: The Guidelines are available at following link: www.swedeye.org/SOTA/rop/SOTA-ROP_2006.pdf.
The evaluation of ROP stage 3 or more will be assessed by retinal examination by a trained ophthalmologist and will be performed in a blinded fashion i.e. the ophthalmologist will be unaware of whether or not the infant is participating in the clinical study.

11.5 Analysis of Safety/Tolerability

Physical examination, MRI and the results of retinal examination will be displayed in per subject listing.

The vital signs and laboratory measurements will be listed per subject and illustrated graphically as levels per time-point (as per Table 1, page 11) by each subject. The measurements will also be summarized by descriptive statistics.

Adverse events will be displayed in per subject listings.

--- ❖ ---

REFERENCES

1. http://www.cdc.gov/nchs/data/nvsr/nvsr64/nvsr64_01.pdf
2. Hodek JM, von der Schulenburg JM, Mittendorf T. Measuring economic consequences of preterm birth - Methodological recommendations for the evaluation of personal burden on children and their caregivers. Health economics review. 2011;1(1):6.
3. Anderson P, Doyle LW, Victorian Infant Collaborative Study G. Neurobehavioral outcomes of school-age children born extremely low birth weight or very preterm in the 1990s. JAMA : the journal of the American Medical Association. 2003;289(24):3264-72.
4. Johnson S, Hollis C, Kochhar P, Hennessy E, Wolke D, Marlow N. Psychiatric disorders in extremely preterm children: longitudinal finding at age 11 years in the EPICure study. Journal of the American Academy of Child and Adolescent Psychiatry. 2010;49(5):453-63 e1.
5. Moreira RS, Magalhaes LC, Alves CR. Effect of preterm birth on motor development, behavior, and school performance of school-age children: a systematic review. J Pediatr (Rio J). 2014;90(2):119-34.
6. Hille ET, Weisglas-Kuperus N, van Goudoever JB, Jacobusse GW, Ens-Dokkum MH, de Groot L, et al. Functional outcomes and participation in young adulthood for very preterm and very low birth weight infants: the Dutch Project on Preterm and Small for Gestational Age Infants at 19 years of age. Pediatrics. 2007;120(3):e587-95.
7. Stoltz Sjostrom E, Lundgren P, Ohlund I, Holmstrom G, Hellstrom A, Domellof M. Low energy intake during the first 4 weeks of life increases the risk for severe retinopathy of prematurity in extremely preterm infants. Arch Dis Child Fetal Neonatal Ed. 2015.
8. Keunen K, van Elburg RM, van Bel F, Benders MJ. Impact of nutrition on brain development and its neuroprotective implications following preterm birth. Pediatr Res. 2015;77(1-2):148-55.
9. EFCNI Benchmarking Report 2009/ 2010 “Too little Too late? Why Europe should do more for preterm Infants” – www.efcni.org
10. McQuire (Ed) ABC of Preterm Birth. Wiley 2009
11. Martin CR, Brown YF, Ehrenkranz RA, O'Shea TM, Allred EN, Belfort MB, et al. Nutritional practices and growth velocity in the first month of life in extremely premature infants. Pediatrics. 2009;124(2):649-57.
12. Beardsall K, Vanhaesebrouck S, Ogilvy-Stuart AL, Vanhole C, Palmer CR, Ong K, et al. Prevalence and determinants of hyperglycemia in very low birth weight infants: cohort analyses of the NIRTURE study. J Pediatr. 2010;157(5):715-9 e1-3.
13. Auerbach A, Eventov-Friedman S, Arad I, Peleg O, Bdolah-Abram T, Bar-Oz B, et al. Long duration of hyperglycemia in the first 96 hours of life is associated with severe intraventricular hemorrhage in preterm infants. J Pediatr. 2013;163(2):388-93.
14. Martin CR, Dasilva DA, Cluette-Brown JE, Dimonda C, Hamill A, Bhutta AQ, et al. Decreased postnatal docosahexaenoic and arachidonic acid blood levels in premature infants are associated with neonatal morbidities. J Pediatr. 2011;159(5):743-9 e1-2.
15. Pawlik D, Lauterbach R, Walczak M, Hurkala J, Sherman MP. Fish-Oil Fat Emulsion Supplementation Reduces the Risk of Retinopathy in Very Low Birth Weight Infants: A Prospective, Randomized Study. JPEN J Parenter Enteral Nutr. 2013.
16. Bernhard W, Raith M, Koch V, Kunze R, Maas C, et al. Plasma phospholipids indicate impaired fatty acid homeostasis in preterm infants. Eur J Nutr. 2014;53:1533-47
17. Simic M, Amer-Wahlin I, Lagercrantz H, Marsal K, Kallen K. Survival and neonatal morbidity among extremely preterm born infants in relation to gestational age based on the last menstrual period or ultrasonographic examination. J Perinat Med. 2014;42(2):247-53.
18. Bernhard W, Raith M, Koch V, Maas C, Abele H Poets CF, et al.Developmental changes in polyunsaturated fetal plasma phospholipids and feto-maternal phospholipid ratios and their association with bronchopulmonary dysplasia. Eur J Nutr 2015.
19. Kurzner SI, Garg M, Bautista DB, Bader D, Merritt RJ, Warburton D, et al. Growth failure in infants with bronchopulmonary dysplasia: nutrition and elevated resting metabolic expenditure. Pediatrics. 1988;81(3):379-84.

20. Martinez M, Ballabriga A. A chemical study on the development of the human forebrain and cerebellum during the brain 'growth spurt' period. I. Gangliosides and plasmalogens. Brain research. 1978;159(2):351-62.
21. Brenna JT. Efficiency of conversion of alpha-linolenic acid to long chain n-3 fatty acids in man. Current opinion in clinical nutrition and metabolic care. 2002;5(2):127-32.
22. Salem N, Jr., Wegher B, Mena P, Uauy R. Arachidonic and docosahexaenoic acids are biosynthesized from their 18-carbon precursors in human infants. Proc Natl Acad Sci U S A. 1996;93(1):49-54.
23. Uauy R, Dangour AD. Nutrition in brain development and aging: role of essential fatty acids. Nutrition reviews. 2006;64(5 Pt 2):S24-33; discussion S72-91
24. German OL, Insua MF, Gentili C, Rotstein NP, Politi LE. Docosahexaenoic acid prevents apoptosis of retina photoreceptors by activating the ERK/MAPK pathway. Journal of neurochemistry. 2006;98(5):1507-20.
25. Clandinin MT, Chappell JE, Leong S, Heim T, Swyer PR, Chance GW. Intrauterine fatty acid accretion rates in human brain: implications for fatty acid requirements. Early Hum Dev. 1980;4(2):121-9.
26. Newman EA. Glial cell regulation of neuronal activity and blood flow in the retina by release of gliotransmitters. Philos Trans R Soc Lond B Biol Sci. 2015:5;370(1672).
27. Bogatcheva NV, Sergeeva MG, Dudek SM, Verin AD. Arachidonic acid cascade in endothelial pathobiology. Microvascular research. 2005;69(3):107-27.
28. Lapillonne A, Jensen CL. Reevaluation of the DHA requirement for the premature infant. Prostaglandins, leukotrienes, and essential fatty acids. 2009;81(2-3):143-50.
29. Zhao JP, Levy E, Fraser WD, Julien P, Delvin E, Montoudis A, et al. Circulating docosahexaenoic acid levels are associated with fetal insulin sensitivity. PLoS One. 2014;9(1):e85054.
30. Connor KM, SanGiovanni JP, Lofqvist C, Aderman CM, Chen J, Higuchi A, et al. Increased dietary intake of omega-3-polyunsaturated fatty acids reduces pathological retinal angiogenesis. Nat Med. 2007;13(7):868-73.
31. Sapieha P, Stahl A, Chen J, Seaward MR, Willett KL, Krah NM, et al. 5-Lipoxygenase Metabolite 4-HDHA Is a Mediator of the Antiangiogenic Effect of {omega}-3 Polyunsaturated Fatty Acids. Science translational medicine. 2011;3(69):69ra12.
32. Stahl A, Sapieha P, Connor KM, Sangiovanni JP, Chen J, Aderman CM, et al. Short communication: PPAR gamma mediates a direct antiangiogenic effect of omega 3-PUFAs in proliferative retinopathy. Circulation research. 2010;107(4):495-500.
33. Pawlik D, Lauterbach R, Turyk E. Fish-oil fat emulsion supplementation may reduce the risk of severe retinopathy in VLBW infants. Pediatrics. 2011;127(2):223-8.
34. Pawlik D, Lauterbach R, Walczak M, Hurkala J, Sherman MP. Fish-Oil Fat Emulsion Supplementation Reduces the Risk of Retinopathy in Very Low Birth Weight Infants: A Prospective, Randomized Study. JPEN J Parenter Enteral Nutr. 2013;38(6):711-6.
35. Beken S, Dilli D, Fettah ND, Kabatas EU, Zenciroglu A, Okumus N. The influence of fish-oil lipid emulsions on retinopathy of prematurity in very low birth weight infants: a randomized controlled trial. Early Hum Dev. 2014;90(1):27-31.
36. Stenson BJ Oxygen saturation targets for extremely preterm infants after the Ne-OProM trials Neonatology 2016;109:352–358.
37. Lau YY, Tay YY, Shah VA, et al., Maintaining optimal oxygen saturation in premature infants, Perm. J. 15 (2011) e108-113.
38. Mills BA, Davis PG, Donath SM, et al., Improving compliance with pulse oximetry alarm limits for very preterm infants, J. Paediatr. Child Health 46 (2010) 255-258.
39. van Zanten HA, Tan RN, van den Hoogen A, et al., Compliance in oxygen saturation targeting in preterm infants: a systematic review, Eur. J. Pediatr. 174 (2015) 1561-1572.
40. Hagadorn JL, Furey AM, Nghiem TH, et al., Achieved versus intended pulse oximeter saturation in infants born less than 28 weeks' gestation: the AVIOx study, Pediatrics 118 (2006) 1574-1582.

41. Manley BJ, Kuschel CA, Elder JE, et al., Higher rates of retinopathy of prematurity after Increasing oxygen saturation targets for very preterm infants: Experience in a single center, J. Pediatr. 168 (2016) 242-244.).

42. James J. Cummings JJ, Polin RA, Committee on fetus and newborn Oxygen Targeting in Extremely Low Birth Weight Infant. Pediatrics 2016;138:e20161576.

43. Henriksen C, Haugholt K, Lindgren M, Aurvag AK, Ronnestad A, Gronn M, et al. Improved cognitive development among preterm infants attributable to early supplementation of human milk with docosahexaenoic acid and arachidonic acid. Pediatrics. 2008;121(6):1137-45.

44. Collins CT, Sullivan TR, McPhee AJ, Stark MJ, Makrides M, Gibson RA. A dose response randomised controlled trial of docosahexaenoic acid (DHA) in preterm infants. Prostaglandins, leukotrienes, and essential fatty acids. 2015;99:1-6.

45. Brans, Y.W.,Andrew, D.S., Carillo, D.W. et al. Tolerance of fat emulsions in very-low-birth-weight neonates. Am. J. Dis. Child. 1988;142:145-52.

46. Folch J, Lees M, Sloane Stanley GH. A simple method for the isolation and purification of total lipides from animal tissues. J Biol Chem. 1957;226(1):497-509.

47. Peng YM, Zhang TY, Wang Q, Zetterstrom R, Strandvik B. Fatty acid composition in breast milk and serum phospholipids of healthy term Chinese infants during first 6 weeks of life. Acta Paediatr. 2007;96(11):1640-5. Epub 2007/10/17.

APPENDIX I

WORLD MEDICAL ASSOCIATION DECLARATION OF HELSINKI

Ethical Principles for Medical Research Involving Human Subjects

Adopted by the 18th WMA General Assembly Helsinki, Finland, June 1964 and amended by the:
29th WMA General Assembly, Tokyo, Japan, October 1975
35th WMA General Assembly, Venice, Italy, October 1983
41st WMA General Assembly, Hong Kong, September 1989
48th WMA General Assembly, Somerset West, Republic of South Africa, October 1996
52nd WMA General Assembly, Edinburgh, Scotland, October 2000

A. INTRODUCTION

1. The World Medical Association has developed the Declaration of Helsinki as a statement of ethical principles to provide guidance to physicians and other participants in medical research involving human subjects. Medical research involving human subjects includes research on identifiable human material or identifiable data.
2. It is the duty of the physician to promote and safeguard the health of the people. The physician's knowledge and conscience are dedicated to the fulfillment of this duty.
3. The Declaration of Geneva of the World Medical Association binds the physician with the words, "The health of my subject will be my first consideration," and the International Code of Medical Ethics declares that, "A physician shall act only in the subject's interest when providing medical care which might have the effect of weakening the physical and mental condition of the subject."
4. Medical progress is based on research which ultimately must rest in part on experimentation involving human subjects.
5. In medical research on human subjects, considerations related to the well-being of the human subject should take precedence over the interests of science and society.
6. The primary purpose of medical research involving human subjects is to improve prophylactic, diagnostic and therapeutic procedures and the understanding of the aetiology and pathogenesis of disease. Even the best proven prophylactic, diagnostic, and therapeutic methods must continuously be challenged through research for their effectiveness, efficiency, accessibility and quality.
7. In current medical practice and in medical research, most prophylactic, diagnostic and therapeutic procedures involve risks and burdens.
8. Medical research is subject to ethical standards that promote respect for all human beings and protect their health and rights. Some research populations are vulnerable and need special protection. The particular needs of the economically and medically disadvantaged must be recognized. Special attention is also required for those who cannot give or refuse consent for themselves, for those who may be subject to giving consent under duress, for those who will not benefit personally from the research and for those for whom the research is combined with care.
9. Research Investigators should be aware of the ethical, legal and regulatory requirements for research on human subjects in their own countries as well as applicable international requirements. No national ethical, legal or regulatory requirement should be

allowed to reduce or eliminate any of the protections for human subjects set forth in this Declaration.

B. BASIC PRINCIPLES FOR ALL MEDICAL RESEARCH

10. It is the duty of the physician in medical research to protect the life, health, privacy, and dignity of the human subject.
11. Medical research involving human subjects must conform to generally accepted scientific principles, be based on a thorough knowledge of the scientific literature, other relevant sources of information, and on adequate laboratory and, where appropriate, animal experimentation.
12. Appropriate caution must be exercised in the conduct of research which may affect the environment, and the welfare of animals used for research must be respected.
13. The design and performance of each experimental procedure involving human subjects should be clearly formulated in an experimental protocol. This protocol should be submitted for consideration, comment, guidance, and where appropriate, approval to a specially appointed ethical review committee, which must be independent of the investigator, the sponsor or any other kind of undue influence. This independent committee should be in conformity with the laws and regulations of the country in which the research experiment is performed. The committee has the right to monitor ongoing trials. The researcher has the obligation to provide monitoring information to the committee, especially any serious adverse events. The researcher should also submit to the committee, for review, information regarding funding, sponsors, institutional affiliations, other potential conflicts of interest and incentives for subjects.
14. The research protocol should always contain a statement of the ethical considerations involved and should indicate that there is compliance with the principles enunciated in this Declaration.
15. Medical research involving human subjects should be conducted only by scientifically qualified persons and under the supervision of a clinically competent medical person. The responsibility for the human subject must always rest with a medically qualified person and never rest on the subject of the research, even though the subject has given consent.
16. Every medical research project involving human subjects should be preceded by careful assessment of predictable risks and burdens in comparison with foreseeable benefits to the subject or to others. This does not preclude the participation of healthy volunteers in medical research. The design of all studies should be publicly available.
17. Physicians should abstain from engaging in research projects involving human subjects unless they are confident that the risks involved have been adequately assessed and can be satisfactorily managed. Physicians should cease any investigation if the risks are found to outweigh the potential benefits or if there is conclusive proof of positive and beneficial results.
18. Medical research involving human subjects should only be conducted if the importance of the objective outweighs the inherent risks and burdens to the subject. This is especially important when the human subjects are healthy volunteers.
19. Medical research is only justified if there is a reasonable likelihood that the populations in which the research is carried out stand to benefit from the results of the research.
20. The subjects must be volunteers and informed participants in the research project.

21. The right of research subjects to safeguard their integrity must always be respected. Every precaution should be taken to respect the privacy of the subject, the confidentiality of the subject's information and to minimize the impact of the study on the subject's physical and mental integrity and on the personality of the subject.
22. In any research on human beings, each potential subject must be adequately informed of the aims, methods, sources of funding, any possible conflicts of interest, institutional affiliations of the researcher, the anticipated benefits and potential risks of the study and the discomfort it may entail. The subject should be informed of the right to abstain from participation in the study or to withdraw consent to participate at any time without reprisal. After ensuring that the subject has understood the information, the physician should then obtain the subject's freely-given informed consent, preferably in writing. If the consent cannot be obtained in writing, the non-written consent must be formally documented and witnessed.
23. When obtaining informed consent for the research project the physician should be particularly cautious if the subject is in a dependent relationship with the physician or may consent under duress. In that case the informed consent should be obtained by a well-informed physician who is not engaged in the investigation and who is completely independent of this relationship.
24. For a research subject who is legally incompetent, physically or mentally incapable of giving consent or is a legally incompetent minor, the investigator must obtain informed consent from the legally authorized representative in accordance with applicable law. These groups should not be included in research unless the research is necessary to promote the health of the population represented and this research cannot instead be performed on legally competent persons.
25. When a subject deemed legally incompetent, such as a minor child, is able to give assent to decisions about participation in research, the investigator must obtain that assent in addition to the consent of the legally authorized representative.
26. Research on individuals from whom it is not possible to obtain consent, including proxy or advance consent, should be done only if the physical/mental condition that prevents obtaining informed consent is a necessary characteristic of the research population. The specific reasons for involving research subjects with a condition that renders them unable to give informed consent should be stated in the experimental protocol for consideration and approval of the review committee. The protocol should state that consent to remain in the research should be obtained as soon as possible from the individual or a legally authorized surrogate.
27. Both authors and publishers have ethical obligations. In publication of the results of research, the investigators are obliged to preserve the accuracy of the results. Negative as well as positive results should be published or otherwise publicly available. Sources of funding, institutional affiliations and any possible conflicts of interest should be declared in the publication. Reports of experimentation not in accordance with the principles laid down in this Declaration should not be accepted for publication.

C. ADDITIONAL PRINCIPLES FOR MEDICAL RESEARCH COMBINED WITH MEDICAL CARE

28. The physician may combine medical research with medical care, only to the extent that the research is justified by its potential prophylactic, diagnostic or therapeutic value. When medical research is combined with medical care, additional standards apply to protect the subjects who are research subjects.

29. The benefits, risks, burdens and effectiveness of a new method should be tested against those of the best current prophylactic, diagnostic, and therapeutic methods. This does not exclude the use of conventional fatty acid supplementation, or no treatment, in studies where no proven prophylactic, diagnostic or therapeutic method exists.
30. At the conclusion of the study, every subject entered into the study should be assured of access to the best proven prophylactic, diagnostic and therapeutic methods identified by the study.
31. The physician should fully inform the subject which aspects of the care are related to the research. The refusal of a subject to participate in a study must never interfere with the subject-physician relationship.
32. In the treatment of a subject, where proven prophylactic, diagnostic and therapeutic methods do not exist or have been ineffective, the physician, with informed consent from the subject, must be free to use unproven or new prophylactic, diagnostic and therapeutic measures, if in the physician's judgment it offers hope of saving life, re-establishing health or alleviating suffering. Where possible, these measures should be made the object of research, designed to evaluate their safety and efficacy. In all cases, new information should be recorded and, where appropriate, published. The other relevant guidelines of this Declaration should be followed.

APPENDIX 2

Appendix 2 - Doseringsschema av Studiepreparat

Studiepreparat	Formulaid™ 2:1 (ARA/DHA)		
	Beredning:	Olja	
	Innehåll:	Arachidonsyra (ARA)<br>Dokosahexaensyra (DHA)	240-290 mg/g (mean 265 mg/g)<br>120-150 mg/g (mean 135mg/g)
	Densitet:	0,9 g/cm^3	

Dosering av Formulaid™

Måldos vid supplementering är: DHA 50 mg/kg/dag samt ARA 100 mg/kg/dag.

Administrering påbörjas vid det 2:a enterala målet efter födelsen och ges om möjligt vid samma tidpunkt varje dag.

Dosökning sker i steg av 0,1 ml enligt separat doseringsschema. Maximal dos är 1 ml/dygn. Dosen beräknas på födelsevikt till dess att den aktuella vikten överskridit födelsevikten och därefter på aktuell vikt.

Enteralt intag	Bröstmjölk och övriga mod-ersmjölksersättningar	Enbart 100% PreNAN Discharge
Formulaid™ dos	0,39 ml/kg/dygn 1 gång dagligen.	0,33 ml/kg/dygn 1 gång dagligen.

	Viktgräns (g) för dosökning	
Formulaid (ml)	Nutrieras med bröstmjölk, övriga ersättningar eller delvis PreNAN Discharge	Nutrieras med enbart med 100% PreNAN Discharge
0,1	260 g	-
0,2	520 g	-
0,3	780 g	-
0,4	1040 g	1215 g
0,5	1300 g	1520 g
0,6	1560 g	1825 g
0,7	1820 g	2130 g
0,8	2080 g	2435 g
0,9	2340 g	2740 g
1,0	2600 g	3045 g

APPENDIX 3

Hantering av studiepreparat:

- När Formulaid-förpackning tinas upp och öppnas fylls 1ml sprutor (för enteral användning) med 1ml Formulaid olja. Sprutorna förvaras i kylskåp tills användning.
- Formulaid dosen ges en gång om dagen oblandad i matsond /oralt före måltid. Sonden spolas genom den efterföljande måltiden med bröstmjölk eller modersmjölksersättning. Ambitionen är att försöka ge Formulaid även om barnet fastar t.ex. p.g.a. retentioner. Ge då Formulaid innan man ger tillbaka retentionen och spola rent sonden med lite luft efteråt. Det är upp till ansvarig kliniker att avgöra om Formulaid kan administreras till det enskilda barnet.
- Under hemsjukvård (när barnen vårdas i hemmet) får föräldrar med sig förfyllda sprutor med viktanpassad dos för en vecka eller fram tills nästa hemsjukvårdsbesök. Vikt kontrolleras minst en gång per vecka.
- Efter utskrivning fortsätter barnet att erhålla förfyllda sprutor till en ålder motsvarande 40 veckor postmenstruell ålder.
- Om patienter flyttas till något annat sjukhus (Ej studie-center) får hemmakliniken ombesörja transport av Formulaid till aktuellt sjukhus, i övrigt rutin enl ovan.

Övrigt

- Mål för full enteral Nutrition: 150-180 ml/kg/dygn
- Multivitamin droppar (Unimedic) ska ges till samtliga barn enligt nedanstående dosering fram tills en uppnådd postmenstruell ålder 40+0 veckor och därefter enligt lokala riktlinjer.

<2 kg	8 droppar
2-3 kg	11 droppar
>3 kg	8 droppar

- PreNAN Discharge är den modersmjölksersättning som i första hand ska användas fram till en uppnådd postmenstruell ålder 40+0 veckor.
