# Supplementary material for: Effect of enteral arachidonic acid and docosahexaenoic acid supplementation on brain volumes at term in preterm infants: a secondary outcome analysis of a randomised controlled trial
Source: Arch Dis Child Fetal Neonatal Ed. 2026 Jan 19;111(4):e328292. doi: 10.1136/archdischild-2024-328292 (PMC13422067; doi:10.1136/archdischild-2024-328292)

Supplement

Effect of Enteral Arachidonic Acid and Docosahexaenoic Acid Supplementation on Brain Volumes at Term in Preterm Infants – A Secondary Outcome Analysis of a Randomized Controlled Trial

William Hellström[1*], Pia Lundgren[2,3], Anders K. Nilsson[2], Staffan Nilsson[4], Anna-Lena Hård[2], Ulrika Sjöbom[2,5], Chatarina Löfqvist[2,5], Isabella M Björkman-Burtscher[6,7], Dirk Wackernagel[8,9], Ingrid Hansen-Pupp[10], Lois EH Smith[11], Boubou Hallberg[12], Karin Sävman[13], David Ley[10], Ann Hellström[2,3], and Rolf A. Heckemann[14]

[1] Department of Pediatrics, Institute of Clinical Sciences, Sahlgrenska Academy, University of Gothenburg, Gothenburg, Sweden

[2] Section for Ophthalmology, Department of Clinical Neuroscience, Institute of Neuroscience and Physiology, Sahlgrenska Academy, University of Gothenburg, Gothenburg, Sweden

[3] Region Västra Götaland, Dept of Pediatric Ophtalmology, The Queen Silvia Children´s Hospital, Sahlgrenska University Hospital, Gothenburg, Sweden

[4] Institute of Biomedicine, Sahlgrenska Academy, University of Gothenburg, Gothenburg, Sweden.

[5] Institute of Health and Care Sciences, Sahlgrenska Academy, University of Gothenburg, Gothenburg, Sweden

[6] Department of Radiology, Institute of Clinical Sciences, Sahlgrenska Academy, University of Gothenburg, Gothenburg, Sweden

[7] Department of Radiology, Section of Neuroradiology, Sahlgrenska University Hospital, Region Västra Götaland, Gothenburg, Sweden

[8] Department of Clinical Science, Intervention and Technology (CLINTEC), Karolinska Institutet, Stockholm, Sweden

[9] Division of Neonatology, Department of Pediatrics, University Medical Center of the Johannes Gutenberg-University Mainz, Mainz, Germany

[10] Department of Clinical Sciences, Lund, Pediatrics, Lund University, and Skåne University Hospital, Lund, Sweden

[11] The Department of Ophthalmology, Boston Children’s Hospital, Harvard Medical School, Boston, MA, USA

[12] Sahlgrenska Academy, University of Gothenburg, Gothenburg, Sweden

[13] Region Västra Götaland, Dept of Neonatology, The Queen Silvia Children´s Hospital, Sahlgrenska University Hospital, Gothenburg, Sweden

[14] Department of Medical Radiation Sciences, Clinical Sciences, Sahlgrenska Academy, University of Gothenburg, Gothenburg, Sweden

*Corresponding author.

W. Hellström, MD, Department of Pediatrics, Institute of Clinical Sciences, The Queen Silvia Children's Hospital, The Sahlgrenska Academy at the University of Gothenburg, S–416 85 Göteborg, SWEDEN. Phone: +46 702465820, Email: william.hellstrom@gu.se

Additional information on nutritional supplementation, randomization, inclusion and ethics

The oil supplement was given enterally together with human milk (mother's own milk or donor milk), preterm formula, or in the buccal cavity at the time of feeding. The randomization was block stratified into three groups according to GA at birth (≤ 24 weeks + 6 days, 25 weeks + 0 days to 26 weeks + 6 days, and 27 weeks + 0 days to 27 weeks + 6 days). Written informed consent was obtained from parents/guardians. The study was conducted in compliance with the Helsinki Declaration.

Intraventricular hemorrhage (IVH) classification

Severe IVH was classified according to modified Papile criteria (IVH grade III; intraventricular hemorrhage exceeding 50% of ventricular volume with signs of acute ventricular distention or IVH grade IV, i.e. periventricular hemorrhagic infarction - PVHI).

Collection of nutritional data

The Nutrium software package (www.nutrium.se, Nutrium AB, Umeå, Sweden) was used for calculation of daily nutrient intakes during postnatal days 2 to 28.

Magnetic resonance imaging and volumetric segmentation

MRI scanning protocol

For volumetry, only T2-weighted images were used after rescaling to 0.8 mm isotropic resolution with sinc interpolation. In Lund, a 2D turbo spin echo (TSE) sequence was used (slice thickness: 3 mm, spacing: 3.6 mm, in-plane resolution ~0.7–0.9 mm, TE: 99 ms, TR: 6,000 ms, flip angle: 150°, echo train length: 18, parallel imaging factor: 2). In Gothenburg, a 3D CUBE T2 sequence was used with 0.8 mm isotropic resolution (TE: 122 ms, TR: 2,752 ms, flip angle: 90°, echo train length: 120, parallel imaging factors: 1.75 in-plane and out-of-plane). In Stockholm, a 2D fast spin echo (FSE) sequence was used (slice thickness: 3 mm, spacing: 3 mm, in-plane resolution ~0.5–0.7 mm, TE: 134 ms, TR: 8,000 ms, flip angle:

115°, echo train length: 16). Head or head–neck coils were used across centers. While most images had a slice thickness of 3 mm, the overall range was 0.8–4 mm, with no systematic variation beyond the protocol differences described above.

MRI examination sedation

For MRI examination, enteral feeding was used for sedation, supplemented, when necessary, by oral chloral hydrate (35 mg/kg) or oral clonidine (2-4 mcg/kg).

MRI segmentation analysis

A senior imaging scientist (R.A.H.), blinded to randomization, reviewed the segmentation output along with the input image stack according to a custom protocol, assigning a quality score to each segmentation. Images where artefacts or other quality deficiencies were judged to compromise volumetric accuracy were excluded. If more than one image stack was available for one and the same participant, the one with the best segmentation quality was retained and the others excluded.

**Supplement Table 1. MRI brain volumes at term equivalent age per study center**		
	**Control**	**AA+DHA intervention**
Center 1	*n=24*	*n=26*
**Total brain volume (cm^3), mean (SD)**	346.2 (36.3)	357.0 (55.8)
**White matter volume (cm^3), mean (SD)**	146.3 (16.0)	150.8 (20.8)
**Cortical grey matter volume (cm^3), mean (SD)**	142.0 (20.6)	147.4 (28.8)
**Central structures volume (cm^3), mean (SD)**	22.5 (2.2)	22.3 (3.1)
**Cerebellum volumes (cm^3), mean (SD)**	26.0 (4.1)	26.8 (5.2)
Center 2	*n=8*	*n=7*
**Total brain volume (cm^3), mean (SD)**	349.3 (18.1)	364.6 (42.9)
**White matter volume (cm^3), mean (SD)**	143.1 (11.2)	156.9 (18.8)
**Cortical grey matter volume (cm^3), mean (SD)**	148.3 (8.2)	149.4 (22.7)
**Central structures volume (cm^3), mean (SD)**	21.6 (1.9)	23.1 (3.3)
**Cerebellum volumes (cm^3), mean (SD)**	27.1 (2.8)	25.9 (3.7)
Center 3	*n=27*	*n=25*
**Total brain volume (cm^3), mean (SD)**	335.2 (36.0)	357.3 (46.8)
**White matter volume (cm^3), mean (SD)**	129.7 (18.0)	141.8 (23.3)
**Cortical grey matter volume (cm^3), mean (SD)**	151.4 (17.8)	159.8 (27.1)
**Central structures volume (cm^3), mean (SD)**	20.5 (2.0)	21.4 (2.0)
**Cerebellum volumes (cm^3), mean (SD)**	25.0 (3.45	25.5 (4.0)
Abbreviations: AA; arachidonic acid, DHA; docosahexaenoic acid, MRI: magnetic resonance imaging, SD; standard deviation		

Supplement Table 2. Infant characteristics of the control and AA+DHA supplemented groups at 36 and 40 weeks PMA				
	**All included infants (n=117)**	**Control (n=59)**	**AA+DHA (n=58)**	**p-value (Controls versus AA+DHA)**
Body weight at 36 PMA weeks, grams, mean (SD)	2384 (331)	2359 (326)	2409 (337)	0.32
Δz-score weight at 36 PMA weeks as compared to at birth, mean (SD)	-0.7 (0.8)	-0.7 (0.8)	-0.6 (0.8)	0.39
Body weight at 40 PMA weeks, MRI, grams, mean (SD)	3150 (410) (n=104)	3125 (398) (n=54)	3176 (426) (n=50)	0.53
Δz-score weight at 40 PMA weeks as compared to at birth, mean (SD)	-0.8 (0.9) (n=104)	-0.8 (0.9) (n=54)	-0.7 (0.9) (n=50)	0.56
Height at birth, cm, mean (SD)	33 (2.4) (n=93)	33 (2.5) (n=45)	33 (2.4) (n=48)	0.81
Z-score height at birth, mean (SD)	-1.3 (1.4) (n=93)	-1.2 (1.5) (n=45)	-1.4 (1.4) (n=48)	0.70
Height at 40 PMA weeks, cm, mean (SD)	47.8 (2.2) (n=102)	47.7 (2.3) (n=54)	48 (2.2) (n=48)	0.47
Z-score height at 40 PMA weeks, mean (SD)	-2.3 (1.3) (n=102)	-2.4 (1.4) (n=54)	-2.2 (1.3) (n=48)	0.39
Head circumference at birth, mean (SD)	23.4 (1.9) (n=114)	23.3 (1.8) (n=57)	23.5 (2.0) (n=57)	0.46
Z-score head circumference at birth, mean (SD)	-0.6 (0.8) (n=114)	-0.6 (0.8) (n=57)	-0.6 (0.8) (n=57)	0.81
Head circumference at 40 PMA	34.6 (1.4) (n=106)	34.4 (1.4) (n=54)	34.8 (1.3) (n=52)	0.16

weeks, cm, mean (SD)				
Z-score head circumference at 40 PMA weeks, mean (SD)	-0.6 (1.0) (n=106)	-0.7 ((1.0) (n=54)	-0.4 (0.9) (n=52)	0.22
Abbreviations: AA; arachidonic acid, DHA; docosahexaenoic acid, GA; gestational age, MRI: magnetic resonance imaging, PMA; post menstrual age, SD; standard deviation				

Supplement Figure 1: Total and subregion brain volumes according to AA+DHA intervention group and controls at term equivalent age stratified by center. a Total brain; **b** White matter; **c** Cortical grey matter; **d** Cerebellum; **e** Central structures. Dots represent individual values where n=24/26 for Control/AA+DHA in Center 1, n=8/7 in Center 2, and n=27/25 in Center 3.

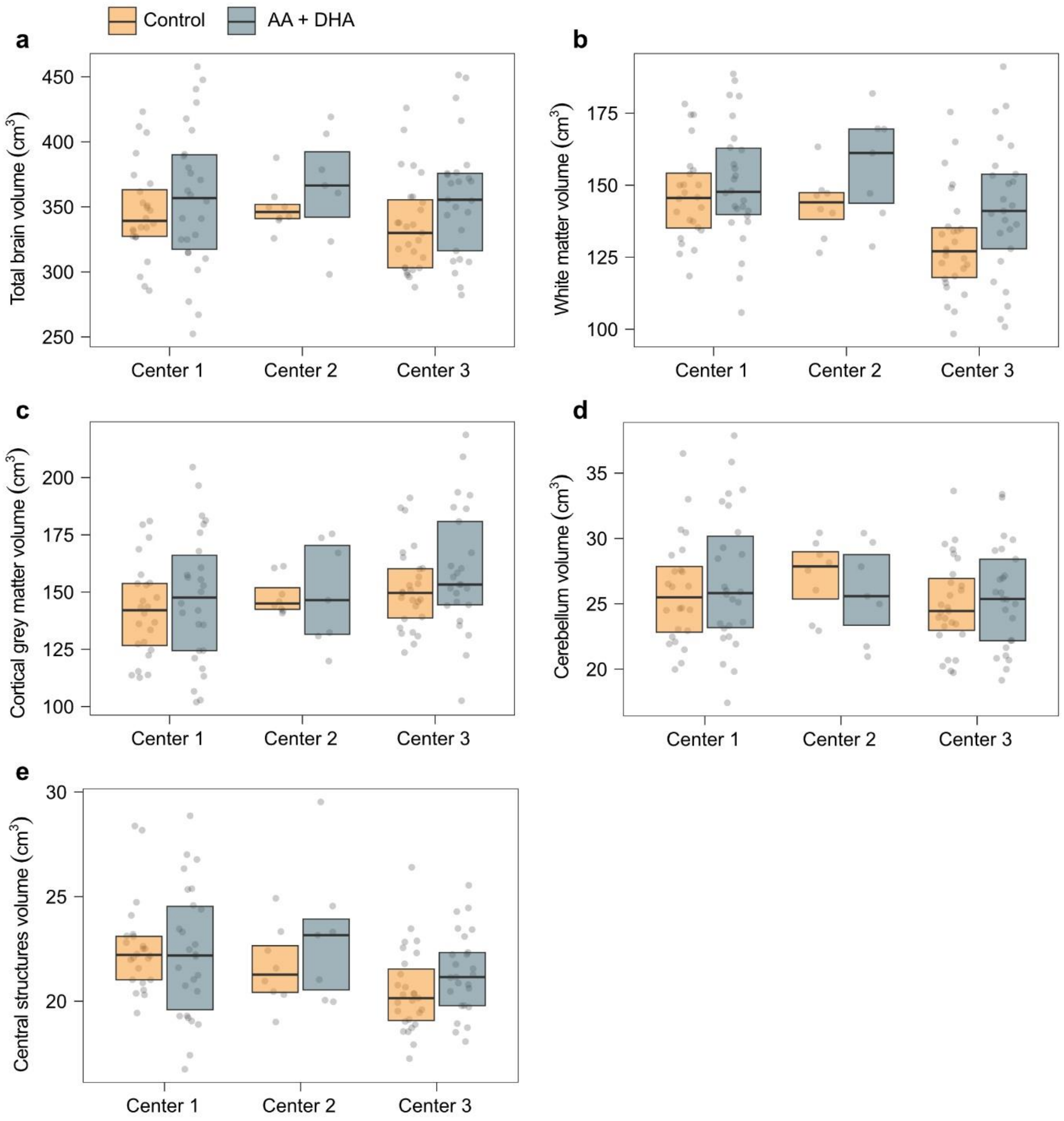

Supplement: online supplemental file 2 [file fetalneonatal-111-4-s002.pdf]
